# Long-read metagenomic sequencing reveals shifts in associations of antibiotic resistance genes with mobile genetic elements from sewage to activated sludge

**DOI:** 10.1186/s40168-021-01216-5

**Published:** 2022-01-29

**Authors:** Dongjuan Dai, Connor Brown, Helmut Bürgmann, D. G. Joakim Larsson, Indumathi Nambi, Tong Zhang, Carl-Fredrik Flach, Amy Pruden, Peter J. Vikesland

**Affiliations:** 1 Department of Civil and Environmental Engineering, Virginia Polytechnic and State University, Blacksburg, VA USA; 2 Department of Genetics, Bioinformatics, and Computational Biology, Virginia Polytechnic and State University, Blacksburg, VA USA; 3 Eawag: Swiss Federal Institute of Aquatic Science and Technology, Kastanienbaum, Switzerland; 4 Institute of Biomedicine, Department of Infectious Diseases, University of Gothenburg, Gothenburg, Sweden; 5 Centre for Antibiotic Resistance Research (CARe), University of Gothenburg, Gothenburg, Sweden; 6 Department of Civil Engineering, Indian Institute of Technology, Madras, India; 7 Department of Civil Engineering, The University of Hong Kong, Hong Kong SAR, China

**Keywords:** Antibiotic resistance, Wastewater, Resistome, Plasmid, Mobile genetic elements, Wastewater treatment, Long read sequencing

## Abstract

**Background:**

There is concern that the microbially rich activated sludge environment of wastewater treatment plants (WWTPs) may contribute to the dissemination of antibiotic resistance genes (ARGs). We applied long-read (nanopore) sequencing to profile ARGs and their neighboring genes to illuminate their fate in the activated sludge treatment by comparing their abundance, genetic locations, mobility potential, and bacterial hosts within activated sludge relative to those in influent sewage across five WWTPs from three continents.

**Results:**

The abundances (gene copies per Gb of reads, aka gc/Gb) of all ARGs and those carried by putative pathogens decreased 75–90% from influent sewage (192-605 gc/Gb) to activated sludge (31-62 gc/Gb) at all five WWTPs. Long reads enabled quantification of the percent abundance of ARGs with mobility potential (i.e., located on plasmids or co-located with other mobile genetic elements (MGEs)). The abundance of plasmid-associated ARGs decreased at four of five WWTPs (from 40–73 to 31–68%), and ARGs co-located with transposable, integrative, and conjugative element hallmark genes showed similar trends. Most ARG-associated elements decreased 0.35–13.52% while integrative and transposable elements displayed slight increases at two WWTPs (1.4–2.4%). While resistome and taxonomic compositions both shifted significantly, host phyla for chromosomal ARG classes remained relatively consistent, indicating vertical gene transfer via active biomass growth in activated sludge as the key pathway of chromosomal ARG dissemination.

**Conclusions:**

Overall, our results suggest that the activated sludge process acted as a barrier against the proliferation of most ARGs, while those that persisted or increased warrant further attention.

Video abstract

**Supplementary Information:**

The online version contains supplementary material available at 10.1186/s40168-021-01216-5.

## Background

Antibiotics are critical for the prevention and treatment of bacterial infections. The spread of antibiotic resistance undermines the effectiveness of antibiotics and is therefore a growing global public health threat [1, 2]. Wastewater treatment plants (WWTPs) receive sewage, including high levels of human pathogens, antibiotics and their metabolites, metals, and other agents that could potentially select or co-select for antibiotic-resistant bacteria (ARB) carrying antibiotic resistance genes (ARGs) [3, 4]. WWTPs thus may play a role in the development and spread of antibiotic resistance [5, 6]. Following physical settling, sewage is typically routed to an activated sludge (AS) basin, an aerated, microbially-rich environment that is among the most widely applied biological treatment practices in the world. AS harnesses microbes to efficiently and economically remove organic contaminants and nutrients, thus protecting aquatic receiving environments. However, there is concern that the AS environment may be fertile ground for ARB propagation and dissemination of ARGs due to high microbial density, diversity, and activity, as well as pressures that might select for ARBs and facilitate ARG transfer [7, 8].

ARGs carried collectively across microbial communities (i.e., resistomes) and individual ARBs have been surveyed in AS and compared to sewage influent at individual WWTPs or multiple WWTPs from the same region using various methods [9–13]. Findings have conflicted with respect to whether AS treatment has the net effect of amplifying [7, 10, 14, 15] or attenuating [16–19] ARGs in the influent. Differences in results may relate to regional variations in sewage resistomes, varying treatment performances among WWTPs, the selected monitoring targets (individual ARGs/ARBs versus full resistomes), or data analysis/normalization approaches [9, 20, 21]. A detailed examination of the fate of sewage-borne ARBs and ARGs across a global transect of representative WWTPs using a consistent methodology is needed to provide insight into the role of the AS process as either a net amplifier of or a barrier to antibiotic resistance dissemination.

Particularly lacking in prior studies is the ability to identify the bacterial carriers of specific ARGs and precise tracking of the fate of specific ARB and ARGs when sewage is introduced into the AS basin. Methodological challenges in obtaining contextual information about ARGs, especially host organisms and co-location with mobile genetic elements (MGEs), which are instrumental in the dissemination of ARGs, have been a barrier to achieving this goal. Culture-based methods can readily track one or a few ARB at a time [16, 22], but are incapable of characterizing entire resistomes [16]. ARG-host and ARG-MGE linkages are often indirectly evaluated via simple correlations or network analysis of contigs assembled from short reads [10, 23, 24]. However, the accuracy of correlation-based analyses is questionable, because the putative relationships identified may be circumstantial or mediated by other unknown or unaccounted for variables and the accuracy of assembly-based methods can be dubious and challenging to verify [25–28].

Nanopore sequencing technology yields long reads from several to hundreds of kilobases. Long reads make it possible to directly evaluate contextual information and determine whether an ARG is located on a plasmid or a chromosome [26, 29]. It is also possible to identify host taxa when the ARG is located on a chromosome, although this is more difficult to determine with certainty when the ARG is located on a plasmid, as these can be hosted by multiple taxa [30–33]. Nanopore sequencing has been more often used for whole genome sequencing of bacterial isolates, with fewer applications toward characterizing highly complex communities [26, 32–35]. One recent study used nanopore metagenomic sequencing of wastewater and provided a useful approach for quantifying ARGs and mapping them to their hosts and/or their genetic context. This study, however, only examined three WWTPs (all in Hong Kong) and did not provide an in-depth comparison of ARG associations with MGEs in influent versus AS [26].

Here, we sought to identify overarching trends as to how the AS process alters raw sewage resistomes by applying nanopore sequencing across five geographically disparate WWTPs located in the USA, Sweden, Switzerland, Hong Kong (China), and India (Additional file: Table S1). Secondly, we sought to evaluate critical contextual information about the identified ARGs, including their genetic location (plasmid or chromosome), co-location with non-plasmid MGEs (transposable elements etc.), and the taxonomy of putative bacterial hosts. The five diverse, globally distributed WWTPs were treated as biological replicates throughout this study to support robust and generalizable conclusions. Overall, the contextual information yielded by nanopore sequencing provides powerful insight into the fate of ARGs and ARBs in sewage subjected to AS treatment.

## Methods

### Sample collection and preparation

Triplicate samples of raw sewage influent (1 L, prior to grit chamber and primary sedimentation) and AS (50 mL, collected at end of aeration stage) were obtained from five globally distributed WWTPs treated as biological replicates (Table S1). Samples were kept on ice and transported to a local laboratory within eight hours of collection. All samples were treated uniformly following the same protocol to support relative comparisons made throughout this study. Biomass in influent samples was concentrated onto 0.22-μm membrane filters and preserved in 50% ethanol before shipment to Virginia Tech in Blacksburg, VA, USA. Aliquots of 0.5-mL AS samples were preserved in an equal volume of 100% ethanol before shipping. DNA was extracted with a FastDNA SPIN kit for soil (MP Biomedicals, Solon OH). Validation of the sample collection, preservation, and DNA extraction approach was previously published [36]. The resulting DNA was purified with a genomic DNA clean kit (Zymo Research, Irvine CA), quantified with a Qubit Fluorometer (ThermoFisher Scientific, Waltham, MA), pooled with equal mass (500 ng) from triplicate samples, and characterized with a NanoPhotometer (Implen, Westlake Village, CA) to examine purity (target OD 260/230 = 2.0–2.2, OD 260/280 > 1.8). If required, further concentration (target > 22 ng/μL) or purification of pooled DNA was conducted using the Zymo genomic DNA clean kit. The absence of degradation during the purification step was confirmed by checking DNA size distribution using DNA Screen Tape (Agilent, Santa Clara, CA).

### Nanopore sequencing

At least 1000 ng DNA was used for each library preparation using the 1D native barcoding genomic DNA kit (SQK-LSK108, EXP-NBD103, Oxford Nanopore Technologies) following the manufacturer’s protocol (v103_21Dec2016). Specifically, DNA fragmentation was conducted with a g-Tube for 1 min at 6000 rpm and quality checked with DNA Screen Tape. A DNA repair step was conducted to fix potential nicks or damage to wastewater DNA. End-prep of fragmented DNA was conducted with an extended incubation of 30 min rather than the recommend 5 min for improved library preparation. Each sample was prepared as an individual library (with barcoding, but no multiplexing) and each was sequenced using a new flow cell (R9.0 or R9.4) in a MinION sequencer. Sequences were collected without real time base calling. Read yields varied among flow cells and library preps, despite efforts to control user-end variables, such as consistent purity and quantity of starting DNA. We set a minimum threshold of collecting 0.6 million reads per sample (post-QC reads > 0.55 million, Table S2) for low-yield flow cells. This minimum sequencing depth was determined based upon a subsampling test using the first batch of four samples (including both influent and AS), demonstrating that the ARG detection rate and composition were relatively consistent across subsampling levels from 0.6 to 3.3 million reads (Additional file: S1). While the extraction procedure used herein did not include a de-circularization step such as that used in some plasmid extractions [37], the mechanical bead-beating lysis step is expected to substantially shear genomic DNA. Furthermore, the N50’s of the raw reads varied between 1.97–6.2 kbp, which should be conducive to recovering fragment plasmids (considering an average size of about 50 kbp [38].

### Sequence analysis

Sequences were base called using Albacore (v2.3.1). Given that all reads from each sequenced library originated from a single sample (i.e., no multiplexing), reads with and without identified barcodes were both used in the subsequent analyses. Reads in fastq files were uploaded to EPI2ME for analysis using the Antimicrobial Resistance Mapping Application (ARMA) pipeline (v3.1.0), which uses the CARD database (v1.1.3) for ARG identification [39]. The built-in cutoff for ARG identification was > 75% nucleic acid alignment identity and > 40% coverage. These criteria are amenable to nanopore data [40], which has a higher intrinsic sequencing error rate (10–15% for R9.4) than Illumina. Similar criteria have been used in other studies [26, 41] and confirmed to have low rates of false positive and false negative for nanopore reads even with simulated higher sequencing error rates up to 45% [42]. ARMA embedded a species identification pipeline, WIMP, which was applied toward phylogenetic identification of all reads [43]. The ARMA pipeline returned all ARG matches detected based on several CARD resistance detection models. Given the high error rate of nanopore reads, only ARG matches identified via the most conservative model—the protein homolog model—were retained. The names and classes of ARGs were manually curated to include updates from the CARD website (accessed April 2019) to accommodate possible errors resulting from the use of an earlier version of the CARD database employed in ARMA.

As defined in Eq. 1, for each ARG, its copy number was calculated as the sum of the alignment coverage over its reference gene for all reads with a match and then normalized to the total base pairs in the sample following quality control (e.g., quality score ≥ 7.0, *Homo sapiens* and virus reads removed). ARG abundance is expressed as the equivalent full length of gene copies per Giga base pair of sequences (gc/Gb) in a sample.

1 $${\mathrm{ARG}}_i\ \mathrm{abundance}\ \left(\mathrm{gc}/\mathrm{Gb}\right)=\frac{\sum_1^m\frac{{\mathrm{Alignment}}_{\mathrm{end}}-{\mathrm{Alignment}}_{\mathrm{start}}}{{\mathrm{Length}}_{\mathrm{reference}}\ \left(\mathrm{bp}\right)}}{\frac{\sum_1^n{\mathrm{Length}}_{\mathrm{read}}\ \left(\mathrm{bp}\right)}{10^9\ \mathrm{bp}/\mathrm{Gb}}}$$

where *i* is the *i*th unique ARG_i_; *m* is the total number of nanopore reads with a match to reference gene(s) for ARG_i_; and *n* is total number of nanopore reads in the sample (post quality screen). The alignment end and start positions, and the length of the reference gene(s) were outputs from ARMA.

Phylogeny was identified by the WIMP module built upon the Centrifuge classifier [44]. This classifier previously demonstrated high accuracy and resolution in microbial profiling from nanopore reads by a recent benchmark study [45] even though it was outperformed by other classifiers for short read application [46]. A taxon ID of the least common ancestor (LCA) was reported for a read when multiple matches were found and was deciphered to the phylogeny according to the U.S. National Institutes of Health guideline [47]. Reads classified as eukaryotic, human (*Homo sapiens*), or viral DNA were excluded. The genome number of a taxon was calculated as the base pair summation of reads classified as this taxon divided by 3.87 Mbp, which is the median genome size among all sequenced bacterial genomes [48].

The location of an ARG-carrying read in a plasmid or chromosome was determined using PlasFlow [49]. We applied a recommended cutoff of 0.7, as suggested by the developers, as we found that significantly fewer reads were available for downstream contextual analysis when higher cutoffs (0.8 or 0.9) were used. Reads shorter than 1000 bp were excluded from PlasFlow per the developer’s suggestion. Phylum classification was also provided by PlasFlow and agreed well with the WIMP outputs (overall 85–99% agreement, 95–99% for chromosome reads). Co-location with MGEs on ARG-carrying reads was analyzed using an in-house developed pipeline NanoARG [42]. NanoARG detects pathogens using NCBI entries corresponding to WHO-identified species [50] as well as the ESKAPE organisms [42].

Phages were annotated using VirSorter [51]. As VirSorter utilizes open reading frames (orfs) that may go undetected due to the high error rate of nanopore data, we also assembled a subset of samples using Canu (with settings corMinCoverage=0, corOutCoverage=all, corMhapSensitivity=high, correctedErrorRate=0.105, genomeSize=5m, corMaxEvidenceCoverageLocal=10, useGrid=false) [52]. To further classify MGEs, a database of bacterial MGE hallmark genes, mobileOG-db [53], was used to assign element class labels of transposable element (defined as sequences derived from ISfinder [54], integrative elements (integrases, transposases, etc, that are not in ISfinder, and which do not encode conjugation machinery), or conjugative element (reads with hits to conjugation machinery). Reads were annotated using DIAMOND [55] at 25% identity and *e* value < 10^−5^_._ This annotation method is analogous to the criteria used by nanoARG [42].

### Statistical analysis

Data analysis was conducted in R (v3.3.3). Nonparametric tests including Wilcox tests (paired, unpaired, or signed test), Dunn test, and Kruskal test were used for comparisons among two or more groups. Proportion tests were applied to test significance in proportions. Non-metric multidimensional scaling (NMDS) multivariate analysis was based on Bray-Curtis dissimilarities in ARG profiles at gene levels, and in community compositions at the most precisely identified taxa, and was complemented with Adonis and ANOSIM analyses. Community correlation was analyzed with Mantel test in the vegan package. Network analysis between ARG class and host phyla was visualized using the circlize and ggraph packages.

## Results and discussion

The Antimicrobial Resistance Mapping Application (ARMA) pipeline (v3.1.0) [56] was applied for ARG identification using its default criteria (> 75% nucleic acid alignment identity and > 40% coverage). These criteria are somewhat less stringent than those typically applied to Illumina data, where amino acid identity (not nucleic acid) is commonly applied at 80–90% [8, 24, 57], but are appropriate for nanopore data given its higher intrinsic sequencing error rate (5–15% vs. 0.1–0.01% in Illumina reads) [26, 41, 42]. Here, we opted for lower, rather than higher, stringency, to maximize the capture of true ARGs, while recognizing that the false positive rate will also likely increase. As the present study relied on relative comparisons of influent vs. AS, the exact cutoffs should not affect the main conclusions, as long as the analysis approach is consistent across samples. No size selection was imposed prior to or within library prep, as we intended to sequence every genetic material in DNA extracts (within which smaller fragments accounted for ~ 15%, Fig. S1).

In total, 377 unique ARGs belonging to 16 classes of antibiotic resistance were detected across all study samples. The ARG detection rate was comparable to previous WWTP studies incorporating Illumina or nanopore sequencing platforms (Table S3). The median alignment accuracies reached upwards of 90%, especially for ARGs located on plasmids (Fig. S2), even though a lower criterion (75%) had been applied. Sequencing depths and total ARG-carrying read base-pairs were also comparable to prior Illumina or nanopore studies (Table S4). Further read details are provided in Additional file: S3.

### Total ARG relative abundance reduced in AS

**Fig. 1:**
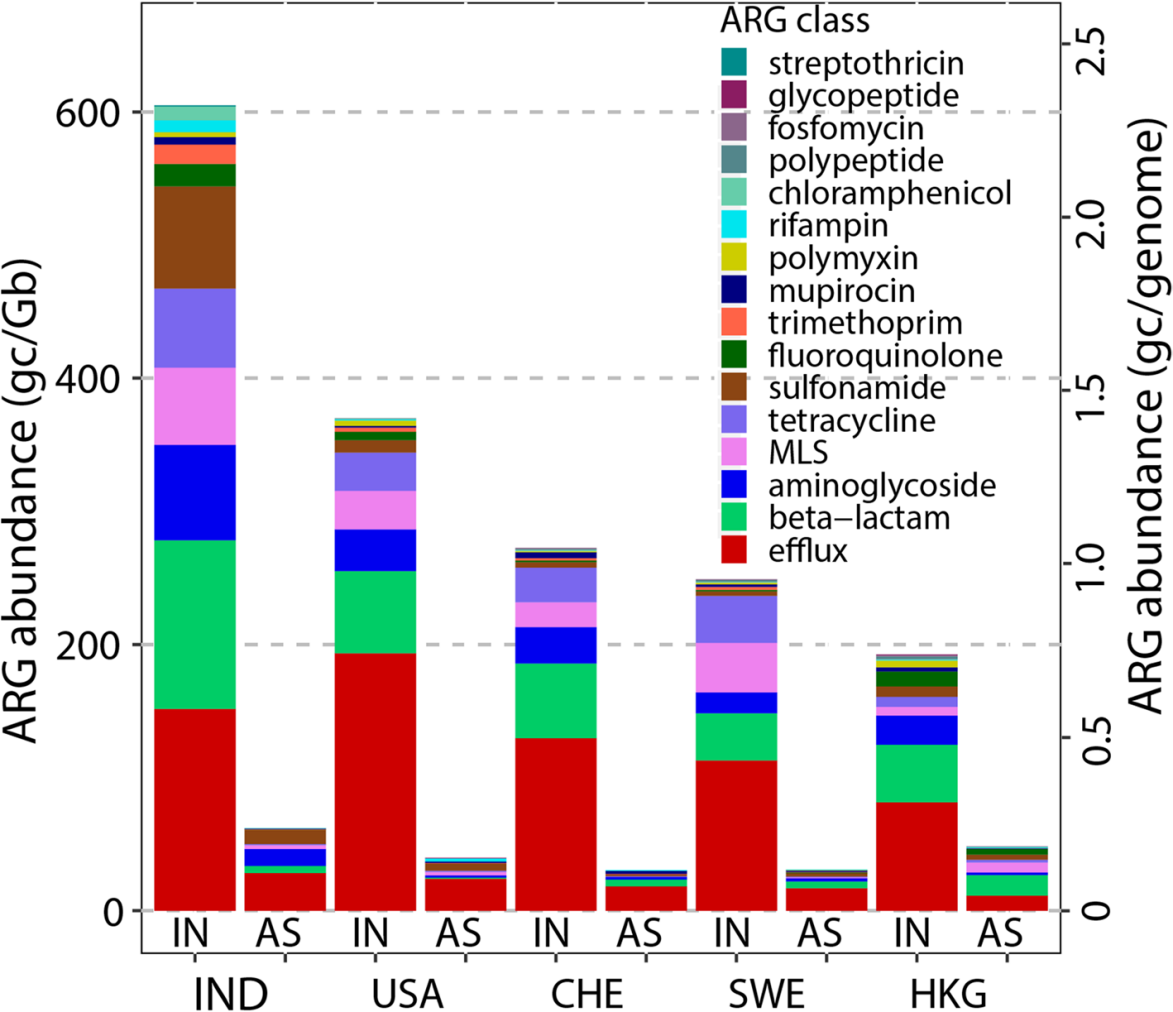
Total ARG abundance decreased from influent (IN) to AS. Five WWTPs are labeled by location: India (IND), The United States (USA), Switzerland (CHE), Sweden (SWE), and Hong Kong (HKG). ARG relative abundance is shown as gene copies per Giga base pairs (gc/Gb), or per sequenced genome (gc/genome) estimated assuming genome sizes of a median value (3.87 Mbp). ARG classes are color-coded and stacked with the most to least abundant from bottom to top

Total ARG relative abundances decreased by 75–90% from sewage influent (192-605 gc/Gb) to AS (31-62 gc/Gb) in all five WWTPs (paired test, *p* = 0.006, Fig. 1), despite differences in geographical factors, pre-AS treatment, and sampling seasons across the transect of WWTPs (Table S1). Notably, the substantial decreases observed were not driven by variable sequencing depths, as there was no correlation between ARG abundance and sequencing depth (Pearson *p* = 0.113). Total ARG relative abundances were also converted to the unit of gc/genome (influent 0.7-2.3 gc/genome, AS 0.1-0.24 gc/genome), by assuming a genome size of 3.86 Mbp (median value among all sequenced bacteria genomes) [48]. Our ARG annotation approach (reduced matching criteria for higher error rate reads) was validated by the results, as our ARG abundances were comparable to prior analyses of sewage (102-605 gc/Gb) and AS (12-945, Table S3) employing different analytical approaches (nanopore, Illumina, high throughput qPCR), relative abundance normalization denominators (16S rRNA genes, Gb, DNA biomass, total number of reads), and ARG identification algorithms and reference databases [10, 24, 26, 57, 58]. Substantial regional variations observed in influent among WWTPs also agree with a recent global sewage monitoring using Illumina sequencing, where samples obtained from Africa and Asia ranked the highest in total ARG abundance [9].

Despite previous concerns about biological treatment enhancing the mobilization and proliferation of ARGs [59], our results provide strong evidence that the AS process does not result in a relative increase of total ARGs among the microbial community (gc/Gb). While a systematic comparison of total ARG abundance from influent to AS across multiple WWTPs like this employed herein has been lacking, prior studies have reported reductions in either individual ARGs [11, 18, 60] or the sum of the many ARGs assayed [23, 24, 58]. A recent study employing nanopore reads indicated a similar reduction (68–92%, calculated from their data) in three Hong Kong WWTPs [26]. Another study indicated an opposite trend of total ARG abundance increasing from influent to AS [10], but this discrepancy may stem from differing sampling locations and comparison points (i.e., raw vs. clarified influent).

We note that the absolute or volumetric abundance (i.e., gc/mL wastewater) may still increase in AS, even though the total ARG relative abundance substantially decreases, because total biomass increases one to three logs between the influent and the AS [17, 18]. Influent is usually mixed with 20–40% of returned AS to promote high bacterial growth rates and to enhance reduction of organic carbon and ammonia. The remaining AS is wasted from the return line each day at a rate proportional to the bacterial growth rate. Thus, reduction in total ARG relative abundance in AS reflects an overall out-competition of incoming ARBs by the massive growth of non-ARB in AS, while selection within certain species of ARB or for certain classes/types of ARGs can still occur [61].

### Total ARG profiles shifted in AS

**Fig. 2:**
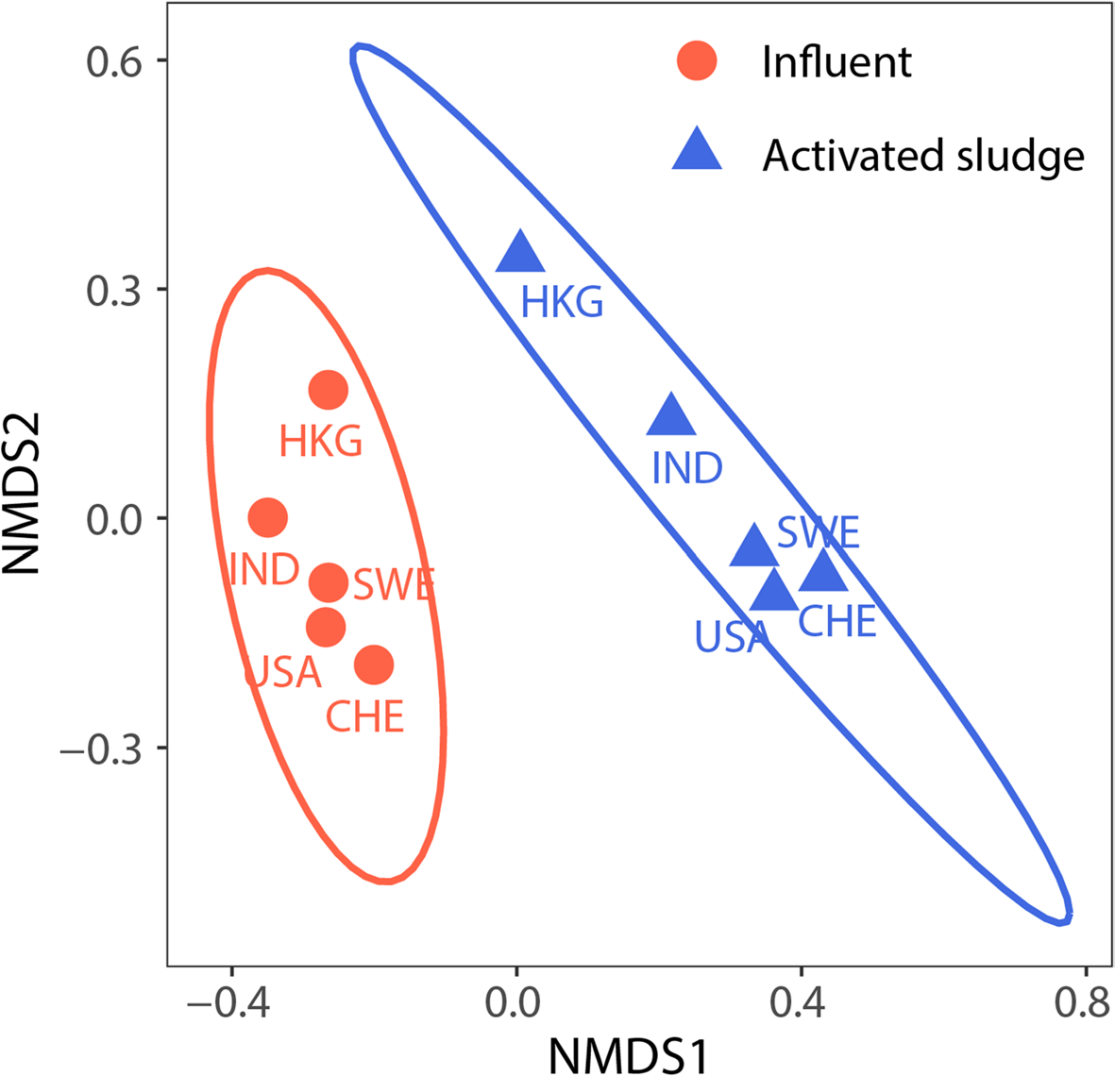
ARG profile shifts from influent to activated sludge (AS). The NMDS plot is based on percent abundances of individual ARGs in influent (red circles) or AS (blue triangles), labeled by location of WWTP: India (IND), The United States (USA), Switzerland (CHE), Sweden (SWE), and Hong Kong (HKG). The ellipses show 95% confidence intervals of influent or AS samples

The resistome composition underwent a significant shift from influent sewage to AS (Fig. 2, Adonis *R*^2^ = 0.38, *p* = 0.001). Influent had more unique ARGs (355, 94% of all detected ARGs, all 16 classes, Fig. 2) than AS (137, 13 classes, Fig. 2). The most abundant ARG classes in influent were multidrug resistance conferred via efflux genes (25–52% of total abundance), beta-lactam (14–23%), and aminoglycoside (6–12%) resistances, similar to previous reports, although the relative ranks and abundances of these classes differed from study to study [10, 57, 62]. In AS, efflux (24–60%) and beta-lactam resistance (2–32%) remained the two most abundant classes, while sulfonamide resistance became the third most abundant class (4–17%) followed by aminoglycoside resistance (4–20%).

Similar clustering of the influent resistome composition away from that of AS was reported for geographically-proximal WWTPs (e.g., in the same plant, city, or country) [10, 11, 23, 58]. Here, the overarching clustering spanning WWTPs across three continents indicated that the shift in ARG profiles from influent sewage to AS is substantial and convergent across WWTPs. This shift further suggests that not all ARGs have the same fate during treatment, rather, some ARGs are more depleted while others are more enriched (these ARGs are detailed further in Additional file: S4).

### Mobility potential of ARGs

#### Genetic location of ARGs varied among ARG classes

The genetic location of an ARG on a plasmid, chromosome, or phage is suggestive of its mobility potential [8, 10, 26]. Herein, the genetic location of an ARG was characterized using PlasFlow [49], while phages were detected using VirSorter (for both raw nanopore reads and on a subset of assembled sequences, Fig. S2). While phages are believed to contribute to ARG spread (e.g., [63]), our analysis detected no ARG-harboring phages in either assembled or raw read data (Fig. S2), and thus these contigs were not analyzed further. Of note, the filter pore sizes (0.22 μm) applied for sample concentration are too large to trap most viral particles, which range in size of prominent features from 10 to 200 nm [64].

**Fig. 3:**
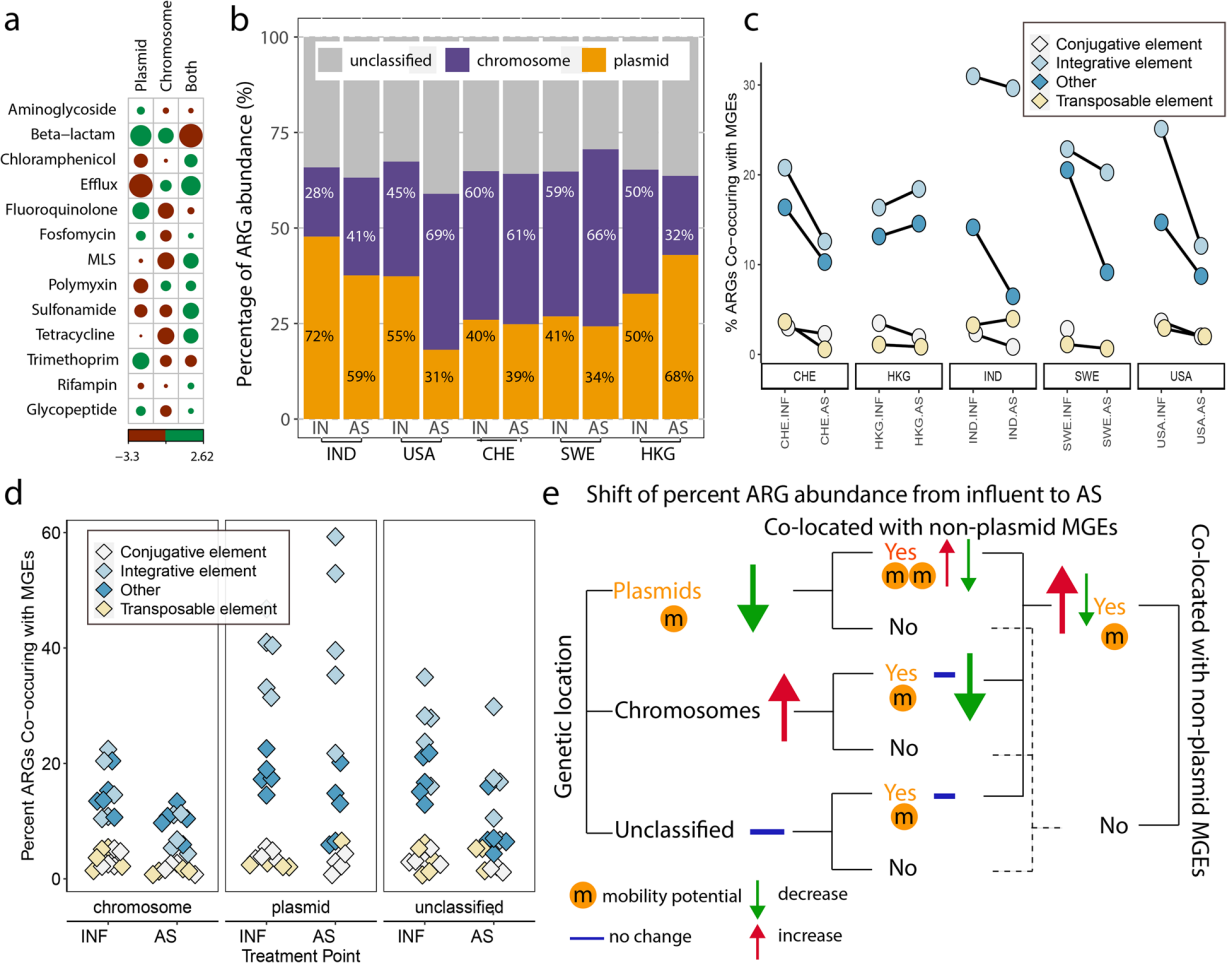
ARG mobility potential. **a** Assignment of unique ARGs to plasmids, chromosomes, or both plasmid and chromosomes across samples varied by ARG classes. Green and maroon circles indicate positive and negative associations, respectively, with larger circles indicating larger correlation residuals. Only classes with more than two unique ARGs were included in the analysis. Percent abundance of ARGs **b** corresponding to different genetic locations; **c** co-located with integrative, transposable, or conjugative element hallmark genes (including transposase, integrase, and/or recombinase genes); or **d** co-located with non-plasmid MGEs, grouped by assigned genetic location on plasmids, chromosomes, or unclassified; **e** Schematic summary of the mobility potential change from influent to AS. While bar charts in panel b included unclassified reads (grey bars), the noted percentages (28%, 72%, etc.) were calculated only among classified reads in a sample, after excluding unclassified reads

In contrast, among the 311 unique ARGs with identifiable genetic locations, 25% (78 ARGs) were assigned as plasmid-encoded, 22% (68 ARGs) only on chromosomes, with the remaining 53% assigned to both plasmids and chromosomes across samples. We observed that the genetic location of a unique ARG was dependent of the class it belongs to (Chi-square *p* < 0.0001, Fig. 3a). More unique beta-lactam ARGs were assigned to either plasmids (*cfxA*, *cfxA5*, etc*.*) or to chromosomes (*cepA*, *cphA4*, *cphA5*, etc.), but fewer were likely to be found on both (*AER*-*1*, *LCR*-*1*, etc.). More unique fluoroquinolone and trimethoprim ARGs were likely to be carried only on plasmids. Relatively more unique ARGs in the sulfonamide, tetracycline, and macrolide-lincosamide-streptogramin MLS classes were assigned to both plasmids and chromosomes, agreeing with reports of their mobility between plasmids and chromosomes [65]. Efflux genes conferring multi-drug resistance were negatively associated with plasmids only, potentially due to increased evolution cost associated with resistance to a wide range of antibiotic classes or as a key feature in many bacteria [66].

#### Percent abundance of ARGs located on plasmids decreased in AS

We further quantified the percent of total ARG abundance in each sample having plasmid-driven mobility potential. Such quantitative analysis was achievable owing to the fact that assembly free reads preserve the counts of the same DNA region (i.e., the coverage for an ARG), which is lost in the assembly process targeting consensus sequences [24]. Where genetic location could be identified (59–71% of total ARG abundance, leaving 29–41% unclassified), putative plasmid-borne ARGs accounted for 40–73% of ARG abundance (excluding unclassified ARG abundance) in influent and 31–68% in AS across the five WWTPs (Fig. 3b). Relative to the influent, the percent abundance of ARGs assigned to plasmids decreased in AS at all WWTPs except Hong Kong (thus non-significant across five WWTPs, *p* = 0.84). This decrease was statistically significant within two WWTPs (IND and USA, *p* < 0.004), which also happened to have the highest percent abundances of plasmid associated ARGs in the sewage influents. Similar decreasing trends were observed within several ARG classes, such as efflux and MLS resistance (Table S5). Notably, plasmid-associated ARGs belonging to beta-lactam and fluoroquinolone resistance classes also tended to decrease in their percent abundance, even though more unique genes within these classes were associated with plasmids (Fig. 3a). Assuming that plasmid-borne ARGs are more likely to transfer horizontally than chromosome-borne ARGs [67], the percent reduction of putative plasmid-borne ARG abundance in AS in four out of five WWTPs is a positive sign that AS reduces the overall potential for horizontal gene transfer [7, 8]. Nonetheless, 31–68% of total ARG relative abundance remains on plasmids in AS and could pose a lingering mobility concern. We could not find other reports on the percent abundance of ARGs being carried on plasmids (not the percent of unique ARGs being on plasmids) from metagenomics samples other than wastewater [26]. This is likely because abundance information is lost when reads are assembled [68] thus restricting prior reports to qualitative descriptions [8, 24].

#### The percent abundance of ARG-harboring MGEs decreased in AS

Co-location with MGE hallmark genes on chromosomes or on plasmids (exemplar MGE reference genes listed in Fig. S3 legend) may be indicative of potential ARG mobility. We found that 33% of 377 unique ARGs detected in this study were co-located on reads encoding hallmarks of integrative, transposable, or conjugative elements. The proportion of ARGs encoded on reads without MGE hallmark genes increased in four of five WWTPs from influent to AS (increases ranging from 9.8 to 18.0%), while, conversely, the proportion of ARGs encoded with integrative, transposable, and conjugative elements decreased or remained at similar levels at these plants in AS (decreases ranging from 0.43–13.0%) (Fig. 3c, Fig. S5). Exceptions to this trend was the Hong Kong WWTP which saw a slight increase in MGE-associated ARGs (1.4–2.4%) and the India WWTP in which ARG-associated transposable elements slightly increased (which increased about 3.5%). These results are consistent with a reduction in plasmid sequences in four of five plants (Fig. 3b) considering plasmids more frequently encoded MGE-associated ARGs than did chromosomal or unclassified sequences (*p* < 0.0001) (Fig. 3d).

When grouping ARGs by their genetic locations, we found that ARG-harboring MGEs were more frequently encoded on plasmids (1–60% abundance) than on chromosomal reads (1–24% abundance) or on unclassified reads (1–45% abundance, *p* < 0.00001, Fig. 3d) for both influent and AS samples. Integrative elements were the most common MGE class (21–60%) found on plasmids. ARGs that are encoded on plasmids or other MGEs likely have elevated mobility, which could enable their persistence and dissemination under selective pressure [69]. Compared to influent samples, we did find a significant decrease in the percent abundance of these genes with elevated mobility potential in AS (*p* < 0.00001, Fig. S4). Together, considering this international transect of WWTPs as biological replicates, our results are not supportive of the hypothesis of an overall selection for or enrichment of ARGs with mobility potential in AS (Fig. 3b,e), although an individual WWTP (e.g., Hong Kong in this study) may exhibit an increased percent abundance for ARGs with plasmid MGE mobility. This result agreed with a prior nanopore study of three Hong Kong WWTPs [26].

The percent of ARGs that co-occurred with MGE (integrative, transposable, and conjugative elements) hallmark genes varied among resistance classes (*p* < 0.0001, Fig. S6). For instance, 37.5% of glycopeptide resistance genes were co-localized with conjugative element hallmark genes, while 28.7% and 29.0% of aminoglycosides and sulfonamide resistance genes, respectively occurred on reads with integrative element hallmark genes. By contrast, efflux pumps were the least frequently found to be co-localized with MGEs (0.41–4.66%). These results support prior observations that some resistance classes have greater potential to be spread horizontally [17, 70].

### ARG hosts: the ARB population

#### ARB represented a distinct subset of the whole bacterial community

Genes neighboring ARGs on the same nanopore reads were analyzed to assess the likely taxonomy of host bacteria. It must be noted that there is inherent uncertainty in such analysis, even with long nanopore reads, because the taxonomic predictive value may vary among neighboring genes [46]. Such assignments are even more uncertain for plasmid reads, because they may be hosted by different taxa [49]. Moreover, available databases of whole genomes and plasmids are limited, and these limitations may introduce bias in the analysis toward what has been deposited in the databases [49]. Bearing these uncertainties in mind, we applied the WIMP module within the ARMA pipeline [56] to predict taxonomic identity for all reads, including those carrying ARGs. This approach assigns the putative taxonomic identity based on the least common ancestor (LCA) in the cases where multiple matches are found for a single read [56].

**Fig. 4:**
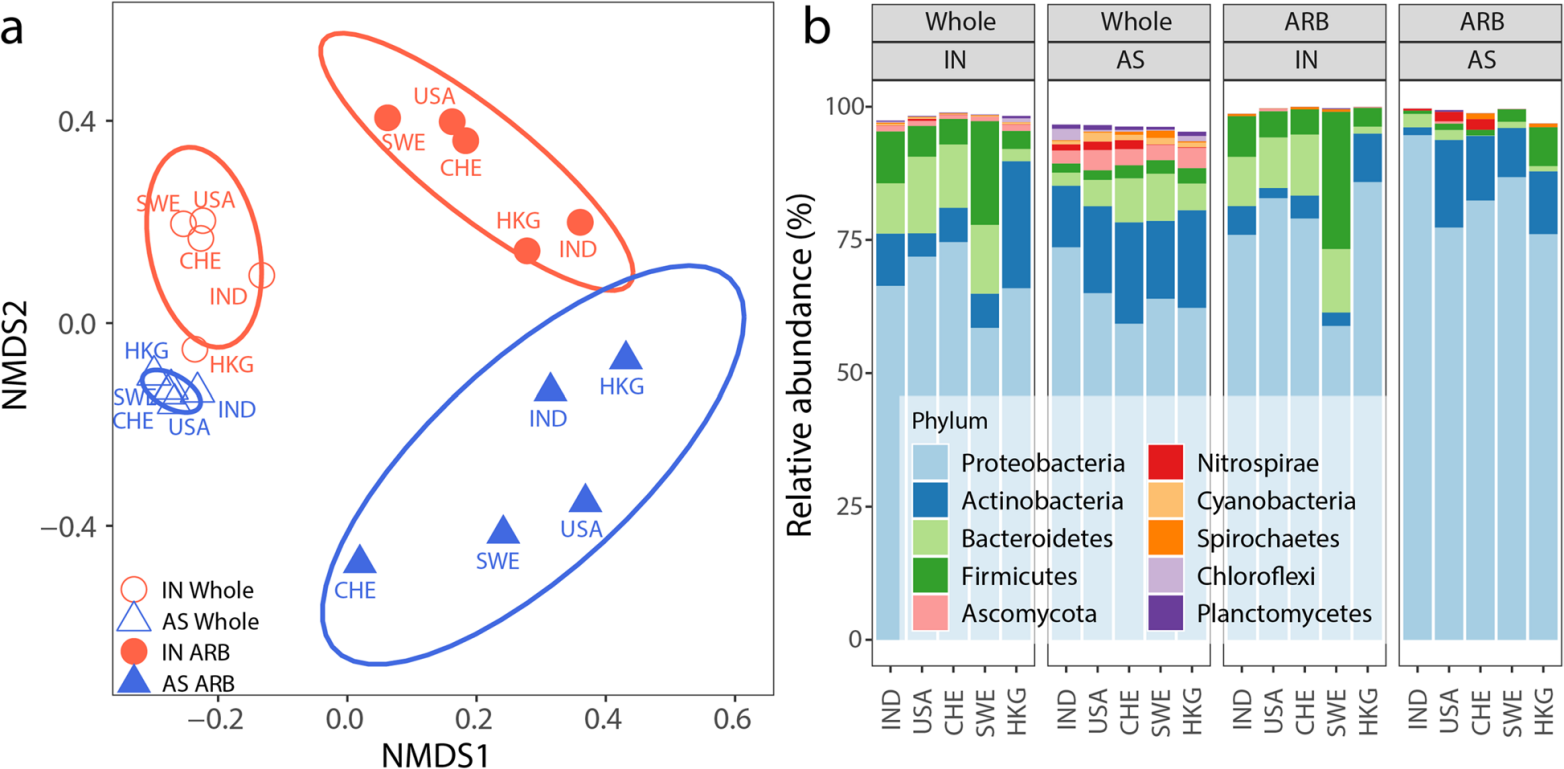
Distinct taxonomic profiles of the bacterial community. Taxonomic profiles of bacterial communities as whole or corresponding subsets of ARB populations were found to be distinct in both the influent (IN) and activated sludge (AS). Bacterial communities as a whole (i.e., all nanopore reads that could be taxonomically classified) and ARB populations (i.e., ARG-carrying reads that could be taxonomically classified, regardless of their genetic location on chromosomes, plasmids, or unidentified) are shown (**a**) clustered separately on an NMDS plot based on taxon abundances and (**b**) as representing distinct proportions of the ten most abundant phyla. The ellipses on panel A indicate 95% confidence interval among samples in a group

The ARB population was consistently distinct in their taxonomic composition relative to that of the whole bacterial community in both influent (ANOSIM *R* = 0.852, *p* = 0.009) and AS (*R* = 0.476, *p* = 0.018, Fig. 4a). ARB population was characterized with 20-fold lower number of taxa (mean 185 vs. 4810, *p* < 0.001), lower Shannon diversity (mean 4.4 vs. 6.5, *p* < 0.001), and higher β-diversity (larger ellipse sizes in Fig. 4a) than the bacterial community as a whole. This result suggests that ARBs were not a random subset of the whole community and that some taxa were more likely to be antibiotic resistant. The ARB population included both abundant (Proteobacteria, Firmicutes, Actinobacteria, and Bacteroidetes) and minor phyla (Fig. 4b), generally agreeing with conclusions from a previous study applying a different methodology (epicPCR) in taxonomy identification of ARGs [62]. While it is not possible from a study of this nature to determine the drivers of the shifts in each taxonomic group, previous research has implicated the stochastic processes of dispersal and drift as well as deterministic factors (such as temperature and organic input) as important parameters in shaping the AS microbial community [20]. For anaerobes in particular (e.g., members of the phyla Bacteroidetes and Firmicutes) the shift from anaerobic to aerobic conditions is likely a key driver.

#### ARB population shifted from influent to activated sludge

The ARB populations in AS were distinct in taxonomic composition relative to those in the influent (ANOSIM *R* = 0.83, *p* = 0.01), even though among-WWTP variations were substantial (mean among-sample similarities is only 25%, Fig. 4a). A notable difference at the phylum level in AS was the decreased relative abundance of Bacteriodetes (which decreased from 8.7 to 1.3%) and Firmicutes (which decreased from 9.3 to 2.5%) in ARBs, compared to influent ARB populations. Bacteriodetes and Firmicutes, both largely anaerobic phyla, are dominant human gut microbiota and are taxa that contain fecal pathogens and ARBs [21, 71]. Their relative abundance in the influent ARB population is consistent with the expectation that fecal bacteria are a primary contributor of ARBs in sewage [72]. Their reduction in AS may result from them being outcompeted by other phyla (e.g., Actinobacteria) that are more adapted to and more actively growing in the richly oxygenated AS environment, which fosters a microbiome more similar to that of freshwater or soil than the human gut [20].

#### Shift in ARG profiles correlates with a shift in the ARB populations and the whole community

The shift of ARG profiles from influent to AS (Fig. 2a) significantly correlated with the compositional changes of the whole bacterial community or the ARB population (Mantel correlation *r* = 0.45–0.47, *p* = 0.002–0.007). Furthermore, host phylum shifts of putatively plasmid-borne or of chromosome-borne ARGs also correlated with changes in the whole bacterial community (*r* = 0.29–0.34, *p* = 0.028 and 0.006, Fig. S7). Host phyla of plasmid and chromosome-associated reads as predicted by PlasFlow [49] agreed with WIMP results, particularly for chromosome-reads (95–99% agreement). Positive correlations between ARG profiles and bacterial community compositions have been previously reported [10]. Consistent with this, our results suggest that not only the resistome, but also the ARB hosts, are closely associated with shifts in the broader bacterial community during the AS process. However, when the correlation analysis was restricted within the same sample type (influent or AS), we found that host phyla of chromosome-borne ARGs, but not of plasmid-borne ARGs (*p* = 0.12–0.5), correlated with the composition of the bacterial community as a whole (*p* = 0.025, *r* = 0.56). This may stem from the fact that host assignments are much more tentative for plasmids than chromosomes. This finding is also expected as plasmid-borne ARGs may occur across multiple species and behave independently of the host, which is known to be the case for many plasmids found in WWTPs [73, 74].

#### Putative pathogenic ARBs decreased in AS

Logically, pathogenic ARBs pose a greater risk to human health than non-pathogenic ARBs. Recognizing that taxonomic resolution obtainable from nanopore reads can be at variable levels, we examined the most precise taxonomic resolution achievable for each read toward identifying putative pathogens. Here, putative pathogens were limited to those classified by the World Health Organization as critical top priority pathogens for which new antibiotics are urgently needed and/or ESKAPE pathogens [50, 75].

We found that the percent abundance of non-plasmid ARGs in putative pathogens significantly decreased from 13–47% in influent to 4–13% in AS in all WWTPs (*p* = 0.005, Fig. S7). Similarly, among ARGs that were detectable in both influent and AS, percent abundance of ARGs hosted by putative pathogens also decreased (7–32% in influent to 2–11% in AS, *p* = 0.03). Note that the analysis here was restricted to the ARGs not encoded on plasmids due to higher uncertainties in taxonomic identification for plasmid sequences. These results suggest that ARB populations shift toward non-pathogenic ARG carriers in AS. This transition may result from decreased numbers of these putative pathogens in AS whole community (Fig. S8), which suggests their less favorable survival in AS environment than other microbes, regardless of carrying an ARG or not. The transition may be also due to ARG transfer from pathogens to non-pathogenic species [76].

### Who carries which ARGs? Additional contextual information yielded from nanopore sequencing

#### Fixed connections between ARG class and host phylum

**Fig. 5:**
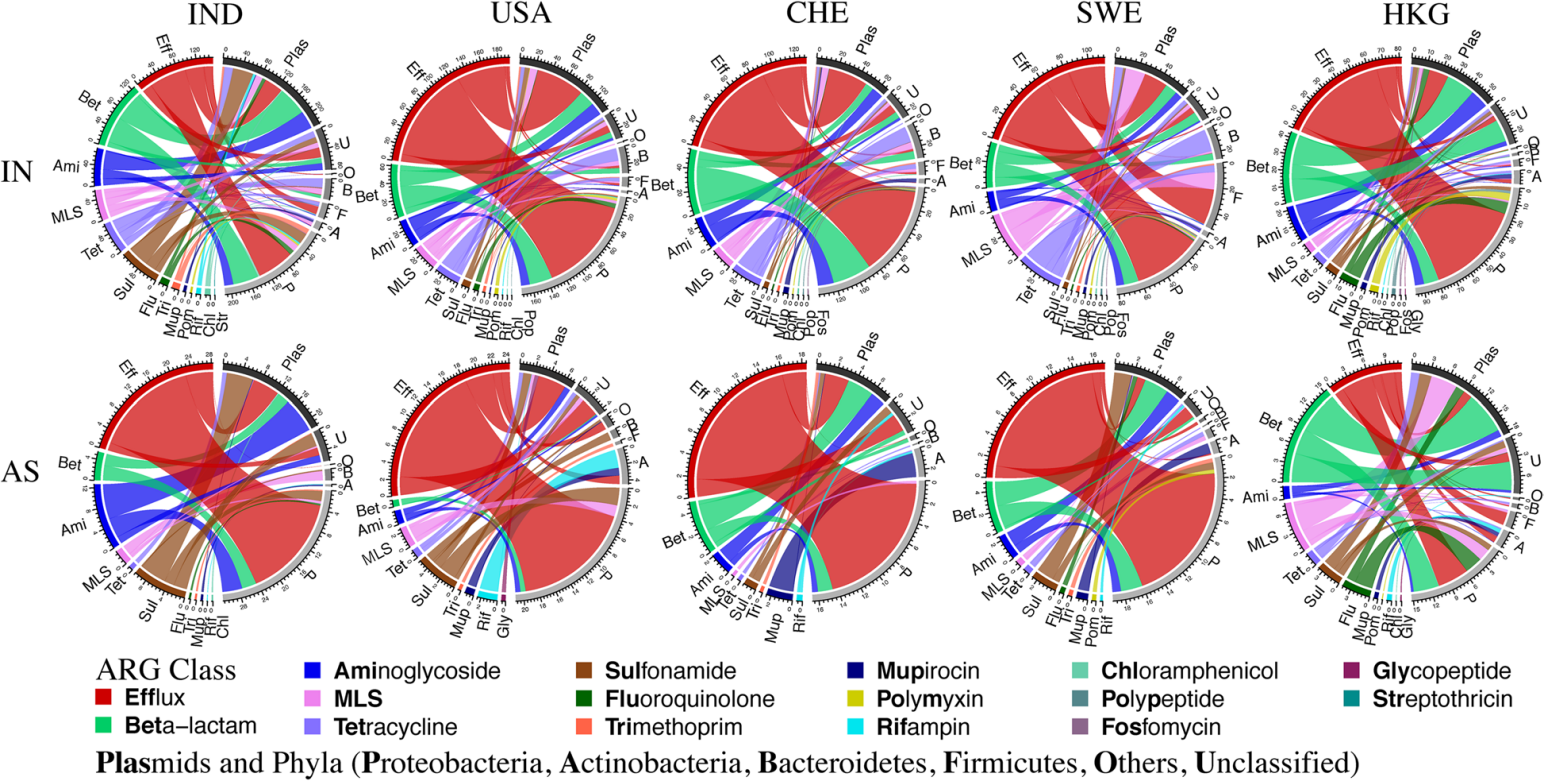
Connections between ARG classes and their putative locations on plasmids or bacteria host phyla. Circular plots are derived from the abundance of ARGs (gc/Gb) in influent (IN- top row) and activated sludge (AS- bottom row) from the five WWTPs. Classes of antibiotic resistance are plotted on the left half of the semi-circles and are color-coded. The assignments of ARG putatively hosted by plasmids or by various phyla are plotted along the right half of the circles and are coded with grey levels

Physical linkages between ARGs (at the class level) and their hosts are illustrated via network analysis in Fig. 5. Here, host phylum is illustrated only for non-plasmid borne ARGs while plasmid-borne ARGs are grouped separately as “plasmid,” because of the higher uncertainty in assigning host taxonomy to plasmids [74, 77]. Although substantial variation was observed among the five WWTPs, a few key patterns were identified for non-plasmid ARGs.

We found that certain ARG classes tended to be preferentially hosted by specific phyla in the influent. For example, the efflux class was hosted predominantly in Proteobacteria (65–92%) in all except Swedish influent, where 32% of efflux abundance was found in Firmicutes. Aminoglycoside resistance was carried mainly in Proteobacteria (60–85%). Beta-lactam resistance was carried by Proteobacteria in all influents (51–75%), except Hong Kong, where 59% was from unclassified taxa. In contrast, tetracycline resistance was carried by Bacteroidetes (22–62%) and Firmicutes (20–27%), yet rarely by Proteobacteria (< 3%). MLS (macrolide-lincosamide-streptogramin) ARGs were more evenly distributed among several phyla, yet 69% of them were carried in Firmicutes in Swedish sewage.

Fixed connections between ARG class and host phyla were also observed in AS. The preferential host phyla of an ARG class is consistent with the understanding that transfer of chromosomal ARGs across phyla is very rare, even though some examples of transfers between Gram positive and Gram negative bacteria have been reported [74].

Contextual taxonomic connections were consistent among ARGs from influent to AS. For example, efflux ARGs and aminoglycoside ARGs were hosted predominantly by Proteobacteria both in influent and AS. Hosts of beta-lactam ARGs remained similar in influent and AS at four WWTPs (mean 46% from Proteobacteria and 30% in plasmids) except in Hong Kong, where 31% was from unclassified reads. This consistency indicates that ARG-host connections were maintained after active biomass growth in the AS process, consistent with vertical transfer being the key pathway for chromosomal ARG dissemination and limited across-phyla horizontal transfer of ARGs. Similarly, horizontal transfer of chromosomal MGEs was reported to be rare across phyla in gut environments [78].

#### Diversity of ARGs hosted by putative pathogens decreased in AS

**Fig. 6:**
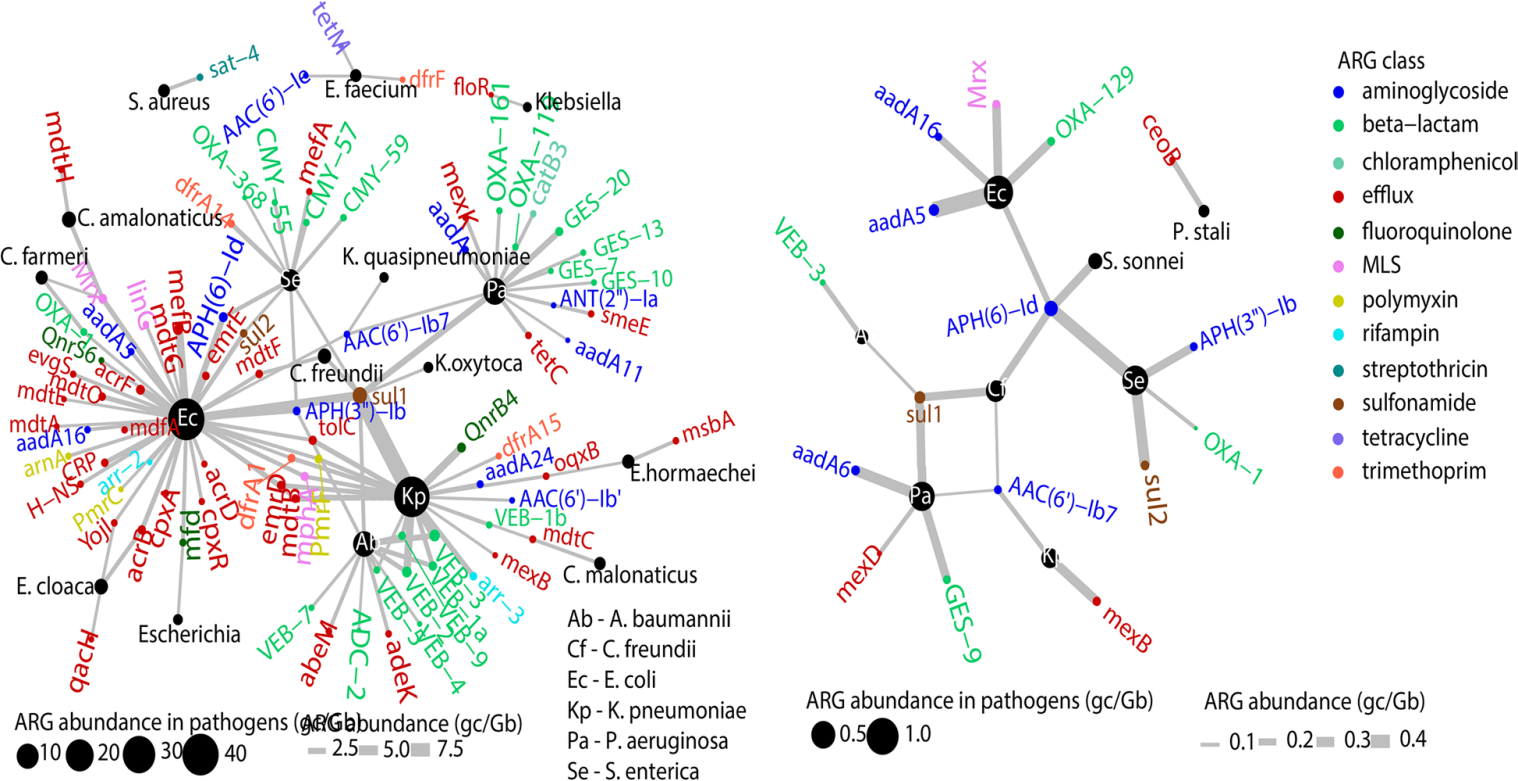
ARGs and their abundances in putative pathogens. Network analysis illustrating ARGs that were carried by putative pathogens (enlisted as critical top priority pathogens by WHO and/or as ESKAPE pathogens) in (**a**) influent (IN) and (**b**) activated sludge (AS) from the Indian WWTP, as an example (network analyses for other WWTPs are available in the SI). The width of edges indicates the abundance of ARGs, and the size of pathogen nodes indicates the summation of ARG abundance hosted by a particular pathogen. Note that different scales of edge width and node size are used in panels (**a**) and (**b**). ARGs are color-coded based on the class to which they belong

As was observed for the other measures discussed above, the diversity and abundance of non-plasmid ARGs hosted by putative pathogens decreased sharply in the AS process in all WWTPs. Multiple best matches for ARGs of the same series (e.g., GES-7, -10, -13, -20, etc.) and the full set of ARGs for efflux pumps were often identified in putative pathogens in influent. Key examples include the beta-lactam ARGs carried by *Pseudomonas aeruginosa* (best matches to 4 GES genes and 2 OXA genes), *Klebsiella pneumoniae* (7 VEB genes), and *Salmonella enterica* (OXA-368 and 3 CMY genes) in Indian influent (Fig. 6), and similarly in other influents (Fig. S9-12). This finding suggests a high genotypic diversity in these putative pathogens that may be beta-lactam or even carbapenem resistant (with best matches to KPC-11, VIM-8). Efflux genes were widely carried by *E*. *coli*, including individual genes (e.g., *mefB*, *emrB*, *emrD*) and operons encoding efflux complexes (e.g., *MdtEF-TolC*, *MdtABC*-*TolC*) and their regulators (e.g., CRP) (Fig. 6, S9-12). *sul1* was found to be hosted by several putative pathogens at high abundances. By contrast, many efflux and beta-lactam ARGs were no longer detected and only two sulfonamide ARGs (*sul1*, *sul2)* and aminoglycoside ARGs (*aadA5,6,16*, APH(6)-Id) remained detectable in AS (Fig. S9-13). Of MGE-associated ARGs detected in both influent and AS samples (*n* = 71), only 10 displayed modest increases (of 1-100%), with no ARG being enriched in more than one WWTP. The rest of the mobile ARGs decreased 3-10,000% in AS (Fig. S14).

Nanopore sequencing provides the ability to contextualize ARGs without the need for assembly, which is a highly complex and error-prone process. Prior work has demonstrated that assembled short reads produced distinct patterns of ARG contextualization compared to long reads, such as those generated by nanopore [79]. Here, nanopore sequencing enabled an assembly-free contextual analysis of samples collected from five WWTPs located on three continents, analyzed as a diverse range of biological replicates, to determine whether AS has a net amplifying or attenuating force on the dissemination of ARGs. Our results suggest that, overall, the AS process acted as a microbial ecological constraint, that attenuated, rather than enriched, the total ARG abundance (normalized to all sequenced nucleotides, gc/Gb) and those carried by putative pathogens in all five WWTPs. Despite differences in geographic, operational, and other parameters, we found that the WWTPs sampled herein had similar shifts in profiles of ARGs (Fig. 1, Fig. 2), MGEs (Fig. 3), and ARBs (Fig. 4A) across each plant. An exception here is that two of five WWTPs (the Hong Kong and India WWTPs) had mixed results in the levels of MGE-associated ARGs from influent to AS. While further work is needed to elucidate the implications, these results do indicate substantial variation in MGE-dynamics in globally distributed sewage and their recipient AS.

Furthermore, a mostly attenuating effect of AS on ARGs, MGEs, and ARG-harboring MGEs was observed across all WWTPs surveyed. This was in part due to a decrease in abundance of ARG-harboring plasmids (40–73% abundance to 31–68%) in four WWTPs. While resistomes and bacterial communities shifted substantially during AS treatment, ARG host phyla for a particular ARG class remained relatively consistent. These results suggest that chromosomal ARGs, even when harbored on MGEs, primarily proliferated as a function of host replication (i.e., cellular growth) in the AS process. It should be noted that metagenomics as utilized here is unable to distinguish between DNA produced from viable and non-viable (dead) cells. Thus, it is unknown whether the putative ARG-harboring reads are derived from viable cells, which may influence the biological interpretation of our results. However, our work is consistent with prior investigations [16] that leveraged exclusively culture-based evidence and found no evidence of selective pressure in the wastewater treatment process.

## Conclusions

This study employs nanopore sequencing to yield quantitative contextual information about ARGs that suggest that the AS process in conventional WWTPs does not create a conducive environment for the proliferation of ARGs or ARBs. However, ARGs associated with MGEs in some individual WWTPs proliferated in AS and may be considered as potential surveillance targets.

## Supplementary Information

**Additional file 1: Fig. S1.** Size and mass distribution of extracted DNA after purification. Measurement was conducted on an Agilent high sensitivity TapeStation. This analysis demonstrated that 13.4% of extracted DNA has fragment smaller than 4851 bp, likely resulted from mechanical cell lysis of bead-beating in the extraction process. **Fig. S2.** VirSorter predicted numerous phages in the raw or assembled nanopore reads. (a). Both raw and assembled nanopore reads produced thousands of viral contigs. Cat1: VirSorter category 1 (most confident phage read/contig); Cat2: Virsorter category 2: (intermediate confidence phage read/contig); cat4: VirSorter category 4 (confident prophages). (b) Taxonomy of the best hits for each mobileOG present on the VirSorter-classified reads. (c) The majority of mobileOG-db hits (290 of 312) had identity values less than 60% and bitscore values under 100. (d) Only four shared mobileOGs were detected (mobileOG_000109022: clpB; chaperone, mobileOG_000314698: insF, integrase; mobileOG_000363469: intF, integrase; mobileOG_000712576: Lambda like terminase). **Fig. S3.** Alignment accuracy (%) of identified ARG sequences in nanopore reads to their reference genes. The alignment accuracy shows the percentage of base pairs in a nanopore read matching the reference ARG. Asterisks indicate significant difference (p<0.01) in alignment accuracies between ARGs located in plasmids and those in chromosomes. **Fig. S4.** The percentage of total number of unique ARGs being co-located with non-plasmid MGEs (including transposase, integrase, or recombinase genes) on the same nanopore reads mostly decreased (<1% - 21.5%). Examples of hallmark genes that were detected include transposase genes include matches with multiple IS family transposase (e.g., IS3, IS5, IS6, IS91, IS1595) from various species, DDE transposase, etc. Integrase genes include integron integrase, site-specific integrase, and *intl1.* Recombinase genes include multispecies recombinase protein family, *tnpR,* etc. “Other” category MGEs refer to matches with mobileOG-db that were not components of integrative, transposable, or conjugative elements. Examples include *repA* and toxin-antitoxin systems, among others. **Fig. S5.** Length (kb) distribution of nanopore reads from influent (IN) and activated sludge (AS) samples from five WWTPs located in India (IND), United States (USA), Switzerland (CHE), Sweden (SWE), and Hong Kong (HKG). **Fig. S6.** Percentage of ARG abundance co-located with hallmarks of transposable, integrative, conjugative and (other) element types. for each ARG class in the ten samples (red Asterix indicate statistically element type-drug class pairs, inferred with the null hypothesis of equal proportions). The percentage was calculated by dividing ARG abundance co-located with non-plasmid MGEs by ARG abundance within a certain ARG class. Box plots shows summary statistics (median, 75 and 25 percentiles, minimum and maximum) in the ten samples. TE: transposable element. **Fig. S7.** Percentage of ARG abundance from different phyla for plasmid-borne or chromosome-borne ARGs in an influent (IN) or activated sludge (AS) sample. Phylum identification for plasmid-borne or chromosome-borne ARGs was based on output from the PlasFlow pipeline, based on host taxonomy of reference plasmids or whole genome databases. The phylum identification agreed with the outputs from the ARMA pipeline. ARG-carrying nanopore reads with no phylum identified were labeled as unclassified. **Fig. S8.** Fate of putative pathogens from influent to AS. (a) Percent abundance of ARGs carried in putative pathogens and (b) percent abundance of these putative pathogens in the whole microbial community (independent of whether the pathogen carries an ARG) in influent and activated sludge samples from the five WWTPs. Putative pathogens were limited to those classified as critical top priority pathogens (e.g., Enterobacteriaceae, *A. baumannii*) by WHO and/or as ESKAPE pathogens. **Fig. S9.** Network analysis illustrating that ARGs were all associated with pathogen-containing taxonomic groups in influent (a) and activated sludge (b) in samples from the WWTP in the United States (USA). **Fig. S10.** Network analysis illustrating that ARGs were all associated with pathogen-containing taxonomic groups in influent (a) and activated sludge (b) in samples from the WWTP in Switzerland (CHE). **Fig. S11.** Network analysis illustrating that ARGs were all associated with pathogen-containing taxonomic groups in influent (a) and activated sludge (b) in samples from the WWTP Sweden (SWE). **Fig. S12.** Network analysis illustrating that ARGs were all associated with pathogen-containing taxonomic groups in influent (a) and activated sludge (b) in samples from the WWTP in Hong Kong (HKG). **Fig. S13.** Individual ARGs that significantly increased (a) or decreased (b) in their abundances (gc/Gb) from influent to AS. Numbers denoted in panel b indicate the median percent reduction in gene abundance from influent to AS. **Fig. S14.** Fate of mobile ARGs across the WWTPs sampled here. Log-fold change in ARG abundance was calculated as the log of the ratio between AS abundance and influent abundance (normalized as gene copies per Gbp). **Fig. S15.** ARG detection rate and profile change with sequencing depth varying from 0.68 to 3.3 million reads. Bar chart by indicates ARG class profile with sequencing depth. MDS plot considers individual ARG abundances. Both influent (IN) and activated sludge (AS) samples from India (IND), Hong Kong (HKG), and Switzerland (CHE) were subsampled to the lowest depth (0.68 million reads), 1.0, half of all reads (1.35, 1.6) and full depth (2.7, and 3.3 million reads). **Table S1.** Sampled WWTPs from five locations. **Table S2.** Nanopore read statistics. **Table S3.** Converted ARG abundances from prior studies* of wastewater treatment plants. **Table S4.** Comparative sequencing depth, counts and base pairs in ARG-carrying reads/contigs identified in this study to exemplar references applying Illumina or ONT sequencing platforms to study wastewater samples from WWTPs. **Table S5.** Percentage of ARG abundance on plasmids for each ARG class and sample location.

## Data Availability

The datasets generated and analyzed during the current study are available at NCBI SRA repository (accession PRJNA628641).

## Abbreviations

ARB: Antibiotic resistant bacteria
ARGs: Antibiotic resistance genes
AS: Activated sludge
ARMA: Antimicrobial Resistance Mapping Application
LCA: Least common ancestor
MGEs: Mobile genetic elements
MLS: Macrolide-lincosamide-streptogramin
NMDS: Non-metric multidimensional scaling
WWTPs: Wastewater treatment plants
