# Supplementary material for: Long-read metagenomic sequencing reveals shifts in associations of antibiotic resistance genes with mobile genetic elements from sewage to activated sludge
Source: Microbiome. 2022 Jan 29;10:20. doi: 10.1186/s40168-021-01216-5 (PMC8801152; doi:10.1186/s40168-021-01216-5)
Supplement: Supplementary file 2 — Additional file 1: Fig. S1. Size and mass distribution of extracted DNA after purification. Measurement was conducted on an Agilent high sensitivity TapeStation. This analysis demonstrated that 13.4% of extracted DNA has fragment smaller than 4851 bp, likely resulted from mechanical cell lysis of bead-beating in the extraction process. Fig. S2. VirSorter predicted numerous phages in the raw or assembled nanopore reads. (a). Both raw and assembled nanopore reads produced thousands of viral contigs. Cat1: VirSorter category 1 (most confident phage read/contig); Cat2: Virsorter category 2: (intermediate confidence phage read/contig); cat4: VirSorter category 4 (confident prophages). (b) Taxonomy of the best hits for each mobileOG present on the VirSorter-classified reads. (c) The majority of mobileOG-db hits (290 of 312) had identity values less than 60% and bitscore values under 100. (d) Only four shared mobileOGs were detected (mobileOG_000109022: clpB; chaperone, mobileOG_000314698: insF, integrase; mobileOG_000363469: intF, integrase; mobileOG_000712576: Lambda like terminase). Fig. S3. Alignment accuracy (%) of identified ARG sequences in nanopore reads to their reference genes. The alignment accuracy shows the percentage of base pairs in a nanopore read matching the reference ARG. Asterisks indicate significant difference (p<0.01) in alignment accuracies between ARGs located in plasmids and those in chromosomes. Fig. S4. The percentage of total number of unique ARGs being co-located with non-plasmid MGEs (including transposase, integrase, or recombinase genes) on the same nanopore reads mostly decreased (<1% - 21.5%). Examples of hallmark genes that were detected include transposase genes include matches with multiple IS family transposase (e.g., IS3, IS5, IS6, IS91, IS1595) from various species, DDE transposase, etc. Integrase genes include integron integrase, site-specific integrase, and intl1. Recombinase genes include multispecies recombinase protein family, [file 40168_2021_1216_MOESM2_ESM.docx]

**Additional file**

**Nanopore sequencing reveals fate of sewage-borne chromosome- and plasmid-associated antibiotic resistance genes in activated sludge**

Dongjuan Dai^1^, Helmut Bürgmann^2^, D.G. Joakim Larsson^3,4^, Indumathi Nambi^5^, Tong Zhang^6^, Carl-Fredrik Flach^3,4^, Amy Pruden^1,*^, and Peter J. Vikesland^1,*^

1. Department of civil and environmental engineering, Virginia Polytechnic and State University, Blacksburg, VA, USA
2. Eawag: Swiss Federal Institute of Aquatic Science and Technology, Switzerland
3. Department of Infectious Diseases, Institute of Biomedicine, Sahlgrenska Academy, University of Gothenburg, Sweden
4. Centre for Antibiotic Resistance Research (CARe), University of Gothenburg, Sweden
5. Department of Civil Engineering, Indian Institute of Technology, Madras, India
6. Department of Civil Engineering, The University of Hong Kong, Hong Kong

*Co-Corresponding authors: [apruden@vt.edu](mailto:apruden@vt.edu), [pvikes@vt.edu](mailto:pvikes@vt.edu)

**Additional Results**

***S1. Minimum sequencing depth***

The first sequenced four samples among the ten were used to evaluate the change of ARG detection rate and ARG profile with sequencing depth. The number of ARG-carrying reads showed a linear increase with the accumulative number of reads sequenced (Fig. S13), suggesting that the rate of detecting ARG did not change with sequencing depth (or sequencing time). Samples were subsampled to the lowest depth of the four samples (0.68 million reads), 1.0 million reads, and half (1.35, 1.6) and full sequencing depth (2.7, 3.3). ARG profile showed to be highly similar among the sample sample subsampled to various levels, which were significantly different from other samples (p<0.0001, Fig. S13) Thus 0.6 million reads (0.55 million post-QC) was considered as the minimum sequencing depth.

***S2. Nanopore read characteristics***

Nanopore sequencing yielded 0.55-3.0 million reads (1.25-6.07 Gb) per sample, with a maximum length of 52 kb (Table S4). N50 across samples ranged from 1,973 to 6,292 bp, which was still in the range of several kilo-base pair though slightly lower than expected (target fragmentation of 8 kb in library prep). Shorter-than-expected sequencing reads were less likely caused due to bead-beating during DNA extraction process, which resulted size peaked around 14-18 kb (Fig. S1). Nicks in double strand DNA can cause the fall off of single strand DNA molecule from nanopore during sequencing, resulting shorter read length than fragment size of double strand DNA, even though a step of FFPE repair to fix such nicks was already conducted in this study. We found that the length distribution of reads significantly differed between sample types (Fig. S4), with a larger N50 in AS than in influent (Δ=1,200-2,700 bp) from all WWTPs, except Hong Kong. Longer reads may reflect fewer DNA nicks in AS-derived DNA, because the AS microbial community is more actively growing than that of the influent, while similar observation was reported by Che et al. when comparing culture samples to WWTP samples.[[1](#_ENREF_1)]

***S3. ARG alignment accuracy***

The alignment accuracy of ARGs in plasmid reads (mean 81-90%) was significantly higher than that of chromosome-borne ARGs (mean 72-83%) in all samples (p<0.0001), regardless of WWTP location and sample type. This difference is likely due to the incompleteness and imperfect in ARG reference database. ARGs carried in plasmids are likely to be better studied and deposited in reference database due to their spread in multiple species and higher risks of horizontal transfer. Thus genetic variants among plasmid-borne ARGs are better learnt than chromosome-borne ARGs. When comparing samples to samples, no significant difference was observed among the five WWTPs in alignment accuracies of either plasmid-borne or chromosome-borne ARGs after controlling for sample type (p>0.20). It suggested the validity of conducting comparisons among sample types, as what we presented in the main text, while bearing in mind that different alignment accuracies for plasmid-borne and chromosome-borne ARGs may have different rates of false positives.

***S4. Individual ARGs enriched or persisting in AS***

Three ARGs (*rph,* *mexY*, and *ceoB*) were enriched in AS across WWTPs (p = 0.03, Fig. S12). Genes *ceoB* and *mexY* encode components of efflux pumps such as CeoAB-OpcM, MexXY-OprM and MexXY-OprA, and the presence of *mexY* in AS was confirmed previously.[[2](#_ENREF_2)] Other genes of these efflux pumps, such as *oprM,* were detected in three WWTP influent samples, but not in AS. The increase of these genes is likely not indicative of specific antibiotic resistance activity, but may be due to a general shift in microbial community structure. Gene *rph* encodes a rifampin phosphotransferase and its expression in Actinobacteria confers resistance to rifampin.[[3](#_ENREF_3)] Its enrichment in AS may reflect increased Actinobacteria in the AS ARB population (Fig. 4b). The increased abundances of these genes in AS suggest that they may be enriched in the treatment process and thus more likely to pass through and reach receiving environments. Indeed, rifampin ARGs were widely detected in treated wastewater and receiving river water.[[4](#_ENREF_4),[5](#_ENREF_5)]

Out of 95 ARGs detected in four or more WWTPs, 31 ARGs displayed significantly reduced abundances in AS with varying percentages of reduction (p=0.01-0.03, Fig. S12). Among the relatively less reduced genes were *sul1* (sulfonamide resistance)*, mexK, mexF, mexW, mexQ* and *smeE* (efflux)*, ileS* (mupirocin resistance)*,* and *aac(6’)-Ib7* (aminoglycoside resistance)*. sul1* (and *sul2*) was previously reported as the least reduced ARG among six others (*qnrS, bla_CTX-M_, bla_OXA-A_, bla_TEM_, bla_SHV_,* and *intI1*) following wastewater treatment.[[6](#_ENREF_6)] In fact, *sul1* is increasingly being used as an indicator of ARG inputs from treated wastewater to receiving environments.[[7-9](#_ENREF_7)] The lesser reduced *mex* genes, along with *mexY* (increased abundance), belong to the resistance-nodulation-division (RND) efflux family, which play a critical role in many clinically*-*relevant antibiotic resistant pathogens.[[10](#_ENREF_10),[11](#_ENREF_11)] Given the potential of clinical significance and multidrug resistance, the fate of these ARGs in the treatment steps that follow the AS process warrant examination in future.

**Supplementary Figures and Tables**

***
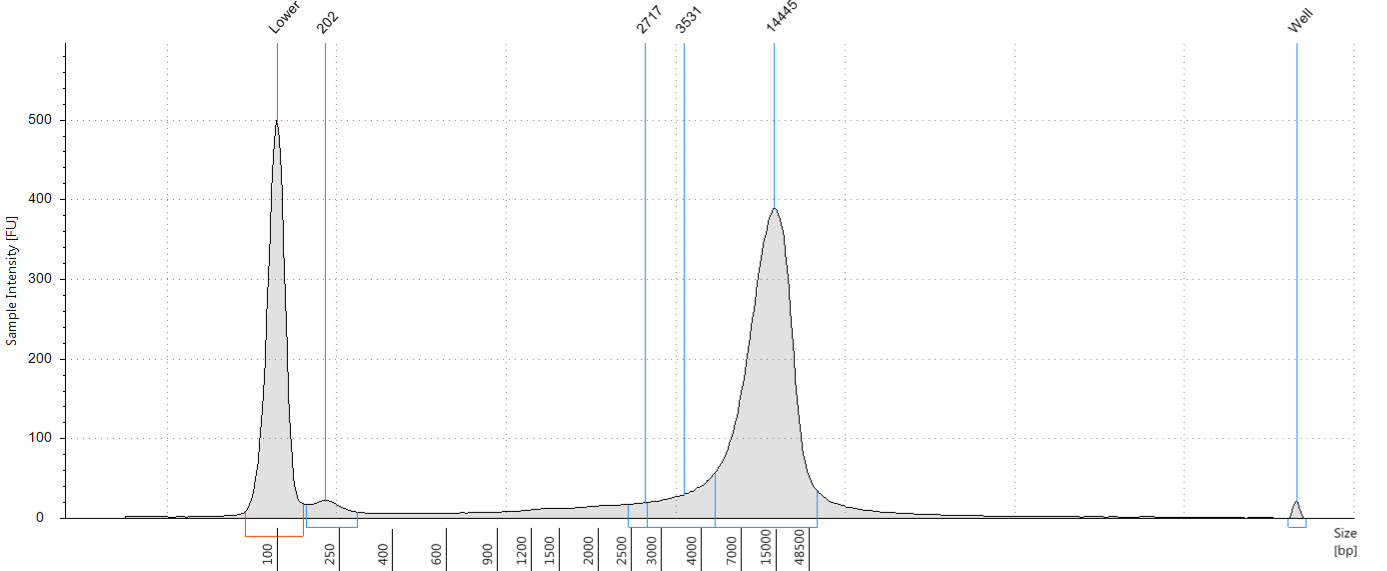
***

| **Fragment size**  **(bp)** | **Mass** |
| --- | --- |
| < 4851 | 13.4 % |
| 4851- 56884 | 86.1 % |

Figure S1. Size and mass distribution of extracted DNA after purification. Measurement was conducted on an Agilent high sensitivity TapeStation. This analysis demonstrated that 13.4% of extracted DNA has fragment smaller than 4851 bp, likely resulted from mechanical cell lysis of bead-beating in the extraction process.


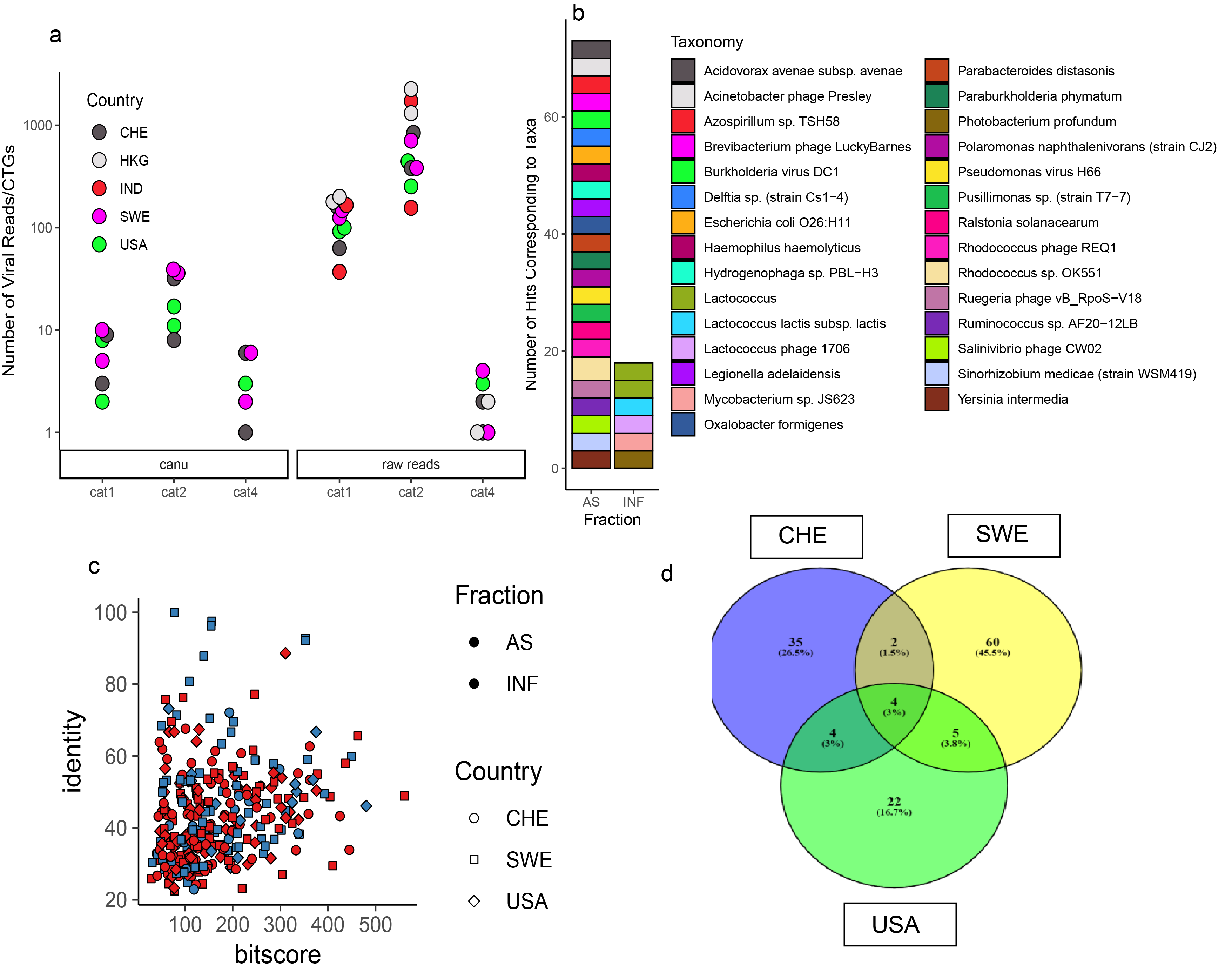


Fig. S2. VirSorter predicted numerous phages in the raw or assembled nanopore reads. (a). Both raw and assembled nanopore reads produced thousands of viral contigs. Cat1: VirSorter category 1 (most confident phage read/contig); Cat2: Virsorter category 2: (intermediate confidence phage read/contig); cat4: VirSorter category 4 (confident prophages). (b) Taxonomy of the best hits for each mobileOG present on the VirSorter-classified reads. (c) The majority of mobileOG-db hits (290 of 312) had identity values less than 60% and bitscore values under 100. (d) Only four shared mobileOGs were detected (mobileOG_000109022: clpB; chaperone, mobileOG_000314698: insF, integrase; mobileOG_000363469: intF, integrase; mobileOG_000712576: Lambda like terminase).

Fig. S3. Alignment accuracy (%) of identified ARG sequences in nanopore reads to their reference genes. The alignment accuracy shows the percentage of base pairs in a nanopore read matching the reference ARG. Asterisks indicate significant difference (p<0.01) in alignment accuracies between ARGs located in plasmids and those in chromosomes.


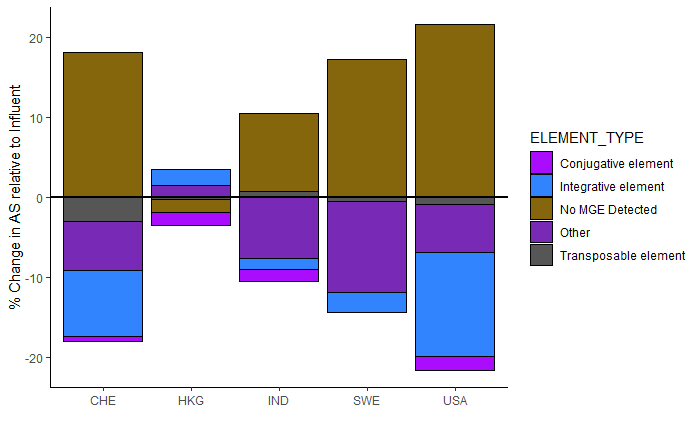


Fig. S4. The percentage of total number of unique ARGs being co-located with non-plasmid MGEs (including transposase, integrase, or recombinase genes) on the same nanopore reads mostly decreased (<1% - 21.5%). Examples of hallmark genes that were detected include transposase genes include matches with multiple IS family transposase (e.g., IS3, IS5, IS6, IS91, IS1595) from various species, DDE transposase, etc. Integrase genes include integron integrase, site-specific integrase, and *intl1.* Recombinase genes include multispecies recombinase protein family, *tnpR,* etc. “Other” category MGEs refer to matches with mobileOG-db that were not components of integrative, transposable, or conjugative elements. Examples include *repA* and toxin-antitoxin systems, among others.

Fig. S5. Length (kb) distribution of nanopore reads from influent (IN) and activated sludge (AS) samples from five WWTPs located in India (IND), United States (USA), Switzerland (CHE), Sweden (SWE), and Hong Kong (HKG).


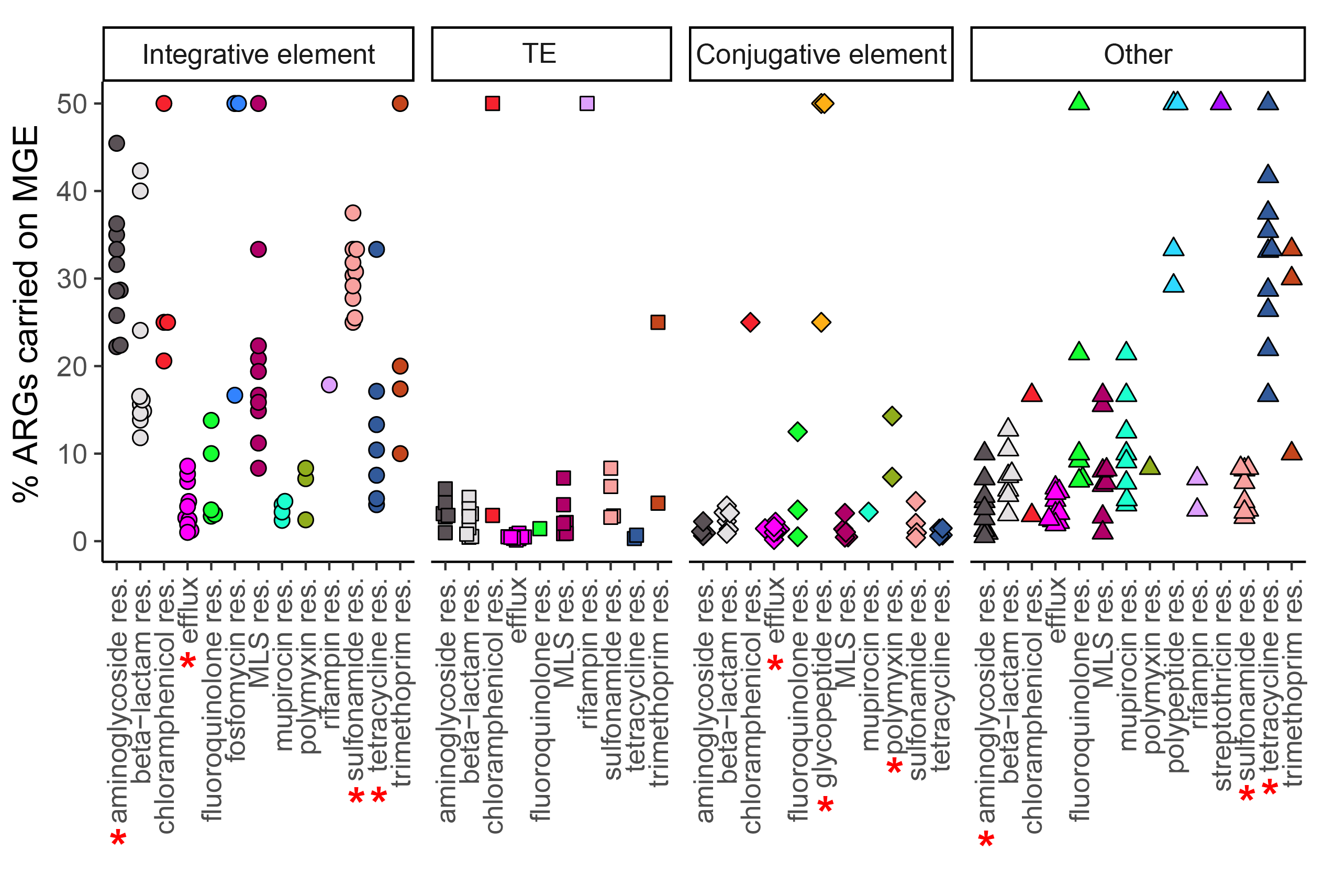


Fig. S6. Percentage of ARG abundance co-located with hallmarks of transposable, integrative, conjugative and (other) element types. for each ARG class in the ten samples (red Asterix indicate statistically element type-drug class pairs, inferred with the null hypothesis of equal proportions). The percentage was calculated by dividing ARG abundance co-located with non-plasmid MGEs by ARG abundance within a certain ARG class. Box plots shows summary statistics (median, 75 and 25 percentiles, minimum and maximum) in the ten samples. TE: transposable element.

Fig. S7. Percentage of ARG abundance from different phyla for plasmid-borne or chromosome-borne ARGs in an influent (IN) or activated sludge (AS) sample. Phylum identification for plasmid-borne or chromosome-borne ARGs was based on output from the PlasFlow pipeline, based on host taxonomy of reference plasmids or whole genome databases. The phylum identification agreed with the outputs from the ARMA pipeline. ARG-carrying nanopore reads with no phylum identified were labeled as unclassified.

Fig. S8. Fate of putative pathogens from influent to AS. (a) Percent abundance of ARGs carried in putative pathogens and (b) percent abundance of these putative pathogens in the whole microbial community (independent of whether the pathogen carries an ARG) in influent and activated sludge samples from the five WWTPs. Putative pathogens were limited to those classified as critical top priority pathogens (e.g., Enterobacteriaceae, *A. baumannii*) by WHO and/or as ESKAPE pathogens.

Fig. S9. Network analysis illustrating that ARGs were all associated with pathogen-containing taxonomic groups in influent (a) and activated sludge (b) in samples from the WWTP in the United States (USA).

Fig. S10. Network analysis illustrating that ARGs were all associated with pathogen-containing taxonomic groups in influent (a) and activated sludge (b) in samples from the WWTP in Switzerland (CHE).

Fig. S11. Network analysis illustrating that ARGs were all associated with pathogen-containing taxonomic groups in influent (a) and activated sludge (b) in samples from the WWTP Sweden (SWE).

Fig. S12. Network analysis illustrating that ARGs were all associated with pathogen-containing taxonomic groups in influent (a) and activated sludge (b) in samples from the WWTP in Hong Kong (HKG).

Fig. S13. Individual ARGs that significantly increased (a) or decreased (b) in their abundances (gc/Gb) from influent to AS. Numbers denoted in panel b indicate the median percent reduction in gene abundance from influent to AS.


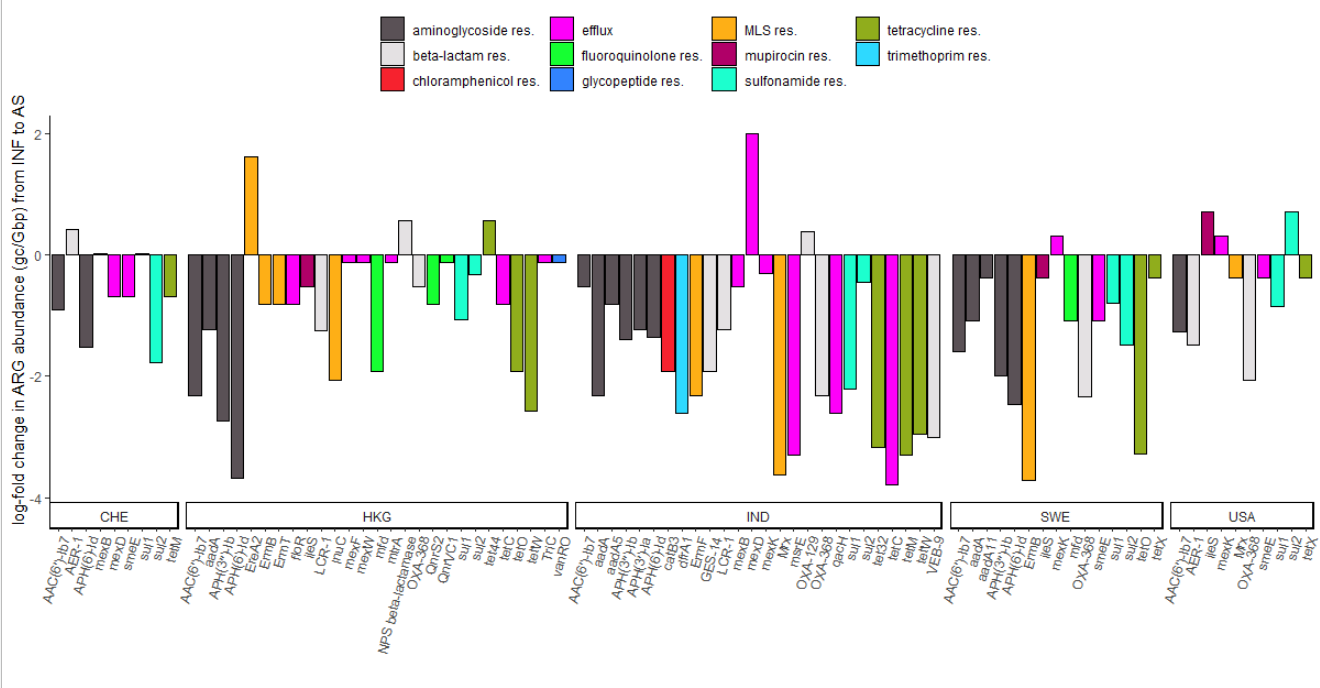


Fig. S14. Fate of mobile ARGs across the WWTPs sampled here. Log-fold change in ARG abundance was calculated as the log of the ratio between AS abundance and influent abundance (normalized as gene copies per Gbp).

|  |  |
| --- | --- |
|  |  |

Figure S15. ARG detection rate and profile change with sequencing depth varying from 0.68 to 3.3 million reads. Bar chart by indicates ARG class profile with sequencing depth. MDS plot considers individual ARG abundances. Both influent (IN) and activated sludge (AS) samples from India (IND), Hong Kong (HKG), and Switzerland (CHE) were subsampled to the lowest depth (0.68 million reads), 1.0, half of all reads (1.35, 1.6) and full depth (2.7, and 3.3 million reads).

Table S1. Sampled WWTPs from five locations

| **WWTP Location** | **Location symbol** | **Wastewater type** | **Treatment capacity** million liters per day (MLD) | **Treatment process** | **Sampling date** |
| --- | --- | --- | --- | --- | --- |
| Hong Kong | HKG | 100% municipal | 250 | Prescreening for solids exceeding 6 mm -> grit removal -> primary sedimentation -> aeration tank (activated sludge) | December 2016 |
| India | IND | 100% municipal | 54 | Two grit chambers -> activated sludge with diffused oxygenators | March 2016 |
| Switzerland | CHE | 90% municipal, 10% industrial | 93 | Grit chamber -> primary sedimentation -> conventional activated sludge | May 2016 |
| Sweden | SWE | Municipal, with some input from industrial and hospital effluent | 25.6^†^ | Grit chamber -> primary sedimentation -> activated sludge (70%) + trickling filter (30%) | June 2016 |
| United States | USA | 95% Municipal, <5% industrial, 0% hospital | 11.4 | Bar screen -> grit chamber -> primary sedimentation ->  conventional activated sludge | November 2016 |

^†^This number reflects the flow on that day.

Table S2. Nanopore read statistics

| **WWTP location** | **Sample type** | **Number of reads** | **Total sequenced base pairs**  **(Gb)** | **Median read length**  **(bp)** | **Maximum read length (bp)** | **Mean  read length  (bp)** | **Minimum read length (bp)** | **Number of reads carrying an ARG  (partial or full)** | **Percentage of reads carrying an ARG  (%)** |
| --- | --- | --- | --- | --- | --- | --- | --- | --- | --- |
| CHE | Influent | 550,203 | 1.59 | 4,707 | 40,552 | 2,897 | 203 | 661 | 0.120% |
| CHE | Activated Sludge | 759,368 | 3.14 | 5,950 | 34,449 | 4,142 | 143 | 249 | 0.033% |
| HKG | Influent | 2,343,436 | 5.34 | 3,868 | 30,167 | 2,279 | 150 | 1797 | 0.077% |
| HKG | Activated Sludge | 2,999,536 | 6.07 | 3,389 | 37,945 | 2,022 | 176 | 622 | 0.021% |
| IND | Influent | 833,396 | 1.25 | 1,973 | 23,723 | 1,496 | 183 | 1224 | 0.147% |
| IND | Activated Sludge | 1,503,839 | 4.26 | 4,600 | 35,804 | 2,834 | 123 | 592 | 0.039% |
| SWE | Influent | 1,068,885 | 2.53 | 3,828 | 34,495 | 2,364 | 210 | 1016 | 0.095% |
| SWE | Activated Sludge | 547,347 | 2.41 | 6,292 | 44,245 | 4,399 | 231 | 220 | 0.040% |
| USA | Influent | 718,617 | 1.38 | 3,211 | 27,885 | 1,919 | 208 | 842 | 0.117% |
| USA | Activated Sludge | 547,303 | 2.03 | 5,973 | 52,276 | 3,715 | 205 | 237 | 0.043% |

Table S3. Converted ARG abundances from prior studies* of wastewater treatment plants

| **Sample type** | **Location of WWTPs** | **Method of measurement** | **Reported ARG abundance in the reference (R)** | **Assumptions used for conversion** | **Conversion formula** | **Converted ARG abundance (gc/Gb)** | **Reference** |
| --- | --- | --- | --- | --- | --- | --- | --- |
| Influent | Hong Kong | ONT sequencing | 255-509 ARG-carrying reads, sequencing depth of 2.5-3.0 Gb | (a) Each ARG-carrying long read has a full gene copy (gc) of the ARG | R/sequencing depth | 102-170 | Che et al. 2019 |
| Influent | Switzerland | Illumina sequencing | Median 0.35 log gc/16S | (b) Genome size of 3.87 Mb, 1 copy of 16S rRNA per genome | 10^R^/3.87x10^-3^Gb | 605 | Ju et al. 2018 |
| Influent | Singapore | Illumina | 1.106 gc/16S | (b) | R/3.87x10^-3^Gb | 298 | Ng et al. 2019 |
| Influent | Finland | High throughput qPCR for 147 ARGs | ~0.045 gc/16S | (b) | R/3.87x10^-3^Gb | 12 | Karkman et al. 2016 |
| Activated Sludge | Hong Kong | Illumina sequencing | 50 ppm (parts per million reads) | (c) Read length 100 bp, ARG full gene size 2000 bp, ARG matching read has 100 bp match to a reference ARG gene | R*100/2000/(1x10^-3^*100)Gb | 25 | Yang et al. 2013 |
| Activated Sludge | Hong Kong | ONT sequencing | 36-87 ARG-carrying reads, sequencing depth of 3.0-5.4 Gb | (a) | R/sequencing depth | 12-32 | Che et al. 2019 |
| Activate Sludge | Taiwan | Illumina sequencing | 3.0-3.5 gc/16S | (b) | R/3.87x10^-3^Gb | 810-945 | Liu et al. 2019 |

*Cited in this table were selected studies of influent or activated sludge samples from municipal wastewater treatment plants from different geographical locations applying various technologies to survey the summation of total ARGs.

Table S4: Comparative sequencing depth, counts and base pairs in ARG-carrying reads/contigs identified in this study to exemplar references applying Illumina or ONT sequencing platforms to study wastewater samples from WWTPs

| **Reference** | **Sequencing platform** | **Sequencing depth (Gb) per sample:** total base pairs in raw reads or in assembled contigs | **Counts of ARG-carrying reads/contigs per sample** | **Calculated total base pairs (kb) in ARG-carrying reads/contigs per sample** |
| --- | --- | --- | --- | --- |
| Che et al. 2019 | ONT | 2.5-3.0 raw reads | 36-478 long reads | 340-5,279 |
| Che et al. 2019 | Illumina | 0.42-0.82 raw reads | 7-83 long contigs | 18-311 |
| Hendriksen et al. 2019 | Illumina | 0.19-28.5 raw reads | 579-55,637 short reads | 87-8,401 |
| Ju et al. 2018 | Illumina | 5.5-6.6 raw reads  0.11-0.29 in contigs used for ARG identification | Average 871 long contigs | 844-8296 |
| Ng et al. 2019 | Illumina | 0.1-0.22 raw reads | Not reported | Not reported |
| Liu et al. 2019 | Illumina | 77-87 raw reads | Not reported | Not reported |
| This study | ONT | 1.3-6.1 raw reads | 220-1,797 long reads | 880-4,095 |

Table S5. Percentage of ARG abundance on plasmids for each ARG class and sample location

| **ARG class** | **Sample type** | **IND (%)** | **USA (%)** | **CHE (%)** | **SWE (%)** | **HKG (%)** |
| --- | --- | --- | --- | --- | --- | --- |
| Aminoglycoside | Influent | 87 | 85 | 54 | 66 | 85 |
| Aminoglycoside (🡹) | Activated sludge | 94 | 85 | 100 | 77 | 86 |
| Beta-lactam | Influent | 87 | 55 | 24 | 45 | 84 |
| Beta-lactam (🡻) | Activated sludge | 83 | NA | 70 | 74 | 83 |
| Chloramphenicol | Influent | 40 | NA | 100 | NA | NA |
| Chloramphenicol (-) | Activated sludge | 100 | NA | NA | NA | NA |
| Efflux | Influent | 47 | 53 | 41 | 27 | 27 |
| Efflux (🡻) | Activated sludge | 31 | 24 | 22 | 7 | 23 |
| Fluoroquinolone | Influent | 84 | 15 | 34 | NA | 66 |
| Fluoroquinolone (🡻) | Activated sludge | NA | NA | NA | 96 | 83 |
| MLS | Influent | 58 | 65 | 71 | 78 | 63 |
| MLS (🡻) | Activated sludge | 16 | 48 | NA | 16 | 92 |
| Mupirocin | Influent | 35 | NA | 32 | 12 | 8 |
| Mupirocin (🡻) | Activated sludge | 36 | NA | 3 | 8 | NA |
| Sulfonamide | Influent | 95 | 100 | 67 | 83 | 67 |
| Sulfonamide (🡻) | Activated sludge | 90 | 67 | 54 | 100 | 75 |
| Tetracycline | Influent | 77 | 35 | 28 | 38 | 35 |
| Tetracycline (🡹) | Activated sludge | 100 | 50 | 100 | NA | 100 |
| Trimethoprim | Influent | 71 | 72 | 51 | 100 | NA |
| Trimethoprim (🡻) | Activated sludge | NA | NA | 100 | NA | NA |

Green: more unique ARGs in this class were preferentially located in plasmids;

Orange: more unique ARGs in this class were located in both plasmids and chromosomes

🡹: increased percentage in Activated Sludge than in influent at three or more WWTPs; 🡻: decreased percentage in three or more WWTPs; and -: lacking data for trend determination

***References***

1 Che Y, Xia Y, Liu L, Li AD, Yang Y ,Zhang T. Mobile antibiotic resistome in wastewater treatment plants revealed by Nanopore metagenomic sequencing. Microbiome 2019; 7: 44.

2 Szczepanowski R, Linke B, Krahn I, Gartemann KH, Gutzkow T, Eichler W *et al.* Detection of 140 clinically relevant antibiotic-resistance genes in the plasmid metagenome of wastewater treatment plant bacteria showing reduced susceptibility to selected antibiotics. Microbiol-Sgm 2009; 155: 2306-2319.

3 Pawlowski AC, Westman EL, Koteva K, Waglechner N ,Wright GD. The complex resistomes of *Paenibacillaceae* reflect diverse antibiotic chemical ecologies. ISME J. 2018; 12: 885-897.

4 Ma LP, Li B ,Zhang T. Abundant rifampin resistance genes and significant correlations of antibiotic resistance genes and plasmids in various environments revealed by metagenomic analysis. Appl. Microbiol. Biotechnol. 2014; 98: 5195-5204.

5 Odjadjare EE, Igbinosa EO, Mordi R, Igere B, Igeleke CL ,Okoh AI. Prevalence of multiple antibiotics resistant (MAR) *Pseudomonas* species in the final effluents of three municipal wastewater treatment facilities in south africa. Int. J. Environ. Res. Public Health 2012; 9: 2092-2107.

6 Narciso-da-Rocha C, Rocha J, Vaz-Moreira I, Lira F, Tamames J, Henriques I *et al.* Bacterial lineages putatively associated with the dissemination of antibiotic resistance genes in a full-scale urban wastewater treatment plant. Environ. Int. 2018; 118: 179-188.

7 Ng C, Tan B, Jiang XT, Gu X, Chen H, Schmitz BW *et al.* Metagenomic and resistome analysis of a full-scale municipal wastewater treatment plant in singapore containing membrane bioreactors. Front. Microbiol. 2019; 10: 172.

8 Pruden A, Arabi M ,Storteboom HN. Correlation between upstream human activities and riverine antibiotic resistance genes. Environ. Sci. Technol. 2012; 46: 11541-11549.

9 Storteboom H, Arabi M, Davis JG, Crimi B ,Pruden A. Tracking antibiotic resistance genes in the South Platte River basin using molecular signatures of urban, agricultural, and pristine sources. Environ. Sci. Technol. 2010; 44: 7397-7404.

10 Li XZ ,Nikaido H. Efflux-mediated drug resistance in bacteria an update. Drugs 2009; 69: 1555-1623.

11 Poole K. Antibiotic Discovery and Development. Vol. 56 (2012).
